# Organelle stresses and energetic metabolisms promote endothelial–to–mesenchymal transition and fibrosis via upregulating FOSB and MEOX1 in Alzheimer’s disease

**DOI:** 10.3389/fnmol.2025.1605012

**Published:** 2025-08-22

**Authors:** Fatma Saaoud, Mohammed Ben Issa, Lu Liu, Keman Xu, Yifan Lu, Ying Shao, Baosheng Han, Xiaohua Jiang, Xiaolei Liu, Avrum Gillespie, Jin Jun Luo, Laisel Martinez, Roberto Vazquez-Padron, Sadia Mohsin, Beata Kosmider, Hong Wang, Silvia Fossati, Xiaofeng Yang

**Affiliations:** ^1^Department of Cardiovascular Sciences, Lewis Katz School of Medicine, Lemole Center for Integrated Lymphatics and Vascular Research, Temple University, Philadelphia, PA, United States; ^2^Department of Cardiovascular Sciences, Lewis Katz School of Medicine, Center for Metabolic Disease Research and Thrombosis Research, Temple University, Philadelphia, PA, United States; ^3^Section of Nephrology, Hypertension, and Kidney Transplantation, Department of Medicine, Lewis Katz School of Medicine, Temple University, Philadelphia, PA, United States; ^4^Department of Neurology, Lewis Katz School of Medicine, Temple University, Philadelphia, PA, United States; ^5^DeWitt Daughtry Family Department of Surgery, Leonard M. Miller School of Medicine, University of Miami, Miami, FL, United States; ^6^Lewis Katz School of Medicine, Aging+Cardiovascular Discovery Center, Temple University, Philadelphia, PA, United States; ^7^Lewis Katz School of Medicine, Center for Inflammation and Lung Research, Alzheimer’s Center, Temple University, Philadelphia, PA, United States; ^8^Lewis Katz School of Medicine, Alzheimer’s Center, Temple University, Philadelphia, PA, United States

**Keywords:** Alzheimer’s disease, endothelial-to-mesenchymal transition (EndoMT), fibrosis, endoplasmic reticulum (ER) stress, cellular stress, metabolic reprogramming

## Abstract

**Introduction:**

Endothelial-to-mesenchymal transition (EndoMT), cell death, and fibrosis are increasingly recognized as contributing factors to Alzheimer’s disease (AD) pathology, but the underlying transcriptomic mechanisms remain poorly defined. This study aims to elucidate transcriptomic changes associated with EndoMT, diverse cell death pathways, and fibrosis in AD using the 3xTg-AD mouse model.

**Methods:**

Using RNA-seq data and knowledge-based transcriptomic analysis on brain tissues from the 3xTg-AD mouse model of AD. This included pathway-level analysis of gene expression changes across multiple brain cell types. Mechanistic insights were further validated using single-cell RNA sequencing (scRNA-Seq) dataset from human AD brain.

**Results:**

Our analysis showed that in the 3xTg-AD model: (i) multiple brain cell type genes are altered, promoting EndoMT through upregulation of RGCC and VCAN; (ii) genes related to various types of cell death, including apoptosis, ferroptosis, necrosis, anoikis, mitochondrial outer membrane permeability programmed cell death, mitochondrial permeability transition-driven necrosis, NETotic, and mitotic cell death, are upregulated in the several brain cell types; (iii) fibrosis-related genes are upregulated across multiple brain cell types. Further mechanistic analysis revealed: (1) mitochondrial stress through upregulation of mitochondrial genes in the brain cells; (2) upregulation of cellular, oxidative, and endoplasmic reticulum (ER) stress genes; (3) nuclear stress via upregulation of nuclear genes, transcription factors (TFs), and differentiation TFs FOSB and MEOX1; (4) metabolic reprogramming/stress through the upregulation of genes related to lipid and lipoprotein metabolism, fatty acid oxidation (FAO), glucose metabolism, and oxidative phosphorylation (OXPHOS); (5) catabolic stress via upregulation of catabolic genes. Single-cell RNA-Seq data indicated that many of these were also increased in AD patients’ brain cells. These changes were reversed by knockdown of the ER stress kinase PERK (EIF2AK3) and deficiencies in FOSB and MEOX1.

**Discussion:**

This study uncovers previously unrecognized molecular signatures of organelle stress and bioenergetic reprogramming that drive EndoMT, cell death, and fibrosis in AD. The reversal of these changes via PERK, FOSB, and MEOX1 inhibition highlights potential therapeutic targets for mitigating neurodegenerative processes in AD.

## 1 Introduction

Alzheimer disease (AD) is a progressive neurodegenerative disorder primarily characterized by memory impairment, cognition decline, and behavioral changes. It is marked by the accumulation of amyloid-beta (Aβ) plaques and tau tangles in the brain, leading to neuronal death and synaptic dysfunction (Knopman et al., 2021; Ricciarelli and Fedele, 2017; Selkoe, 2001; Zhao et al., 2020). As the leading cause of senile dementia, the number of individuals aged 65 and older living with AD in the United States is projected to increase from 5.8 million in 2020 to 13.8 million by 2050 (Zhao et al., 2020). Globally, an estimated 55 million people are affected by AD and other forms of dementia (Alzheimer’s Association, 2024)^1^. Although the exact cause of AD remains unclear, it is believed to result from a combination of genetic, environmental, and lifestyle factors. Mutations in the amyloid precursor protein (APP) and presenilin 1 (PSEN1) genes are linked to familial forms of the disease, while the apolipoprotein E (APOE) gene (Maezawa et al., 2004) is a major genetic risk factor for late-onset AD. Neuroinflammation, vascular dysfunction, and blood-brain barrier (BBB) disruption are also integral components of AD pathology. Despite significant advances in research, effective treatments for AD remain limited, with current therapies primarily focused on alleviating symptoms rather than addressing the underlying causes. Further advancements in understanding AD progression are essential for developing new and effective therapeutics strategies.

In our recent study (Saaoud et al., 2025), RNA-sequencing (RNA-seq) analysis of brain tissue from 3xTg-AD mice, including a volcano plot and heat map, identified 316 upregulated and 412 downregulated genes. These included genes associated with the BBB, cerebrospinal fluid, and proinflammatory markers. The upregulation of genes linked to cell migration, differentiation, and trans-differentiation suggests that inflammation and cellular plasticity may play significant roles in AD pathogenesis. Additionally, genes involved in inflammasome pathways, immunometabolism, and trained immunity (innate immune memory for inflammation enhancement) were upregulated, indicating their potential contribution to AD progression. This study highlighted how trained immunity and inflammasome activation might influence AD development and revealed mechanistic insights that point to potential therapeutic targets for neuroinflammation and cellular reprogramming in AD. However, our previous study did not investigate whether organelle stress and bioenergetic mechanisms underlie cerebrovascular dysfunction and chronic inflammation in the AD brain.

We recently proposed a new concept that pathological trans-differentiation is a novel therapeutic target for cardiovascular diseases and chronic inflammation (Yang et al., 2024). Endothelial-to-mesenchymal transition (EndoMT) is a process where endothelial cells (ECs) lose their properties and differentiate into multipotent mesenchymal cells (Song et al., 2020), which occurs during both development and various pathologies, such as cancer progression, inflammation, and organ/tissue fibrosis (Yoshimatsu and Watabe, 2022). Several signaling pathways known to induce EndoMT (Xiong et al., 2018) are implicated in central nervous system pathologies associated with BBB dysfunction (Derada Troletti et al., 2016), yet transcriptomic reprogramming of the EndoMT pathway in AD remains poorly understood. Additionally, regulated cell death plays a significant role in AD (Thal et al., 2024). In 2018, the International Nomenclature Committee classified 14 types of cell death, including intrinsic and extrinsic apoptosis, mitochondrial permeability transition-driven necrosis, necroptosis (Richard and Mousa, 2022), ferroptosis, pyroptosis (Wójcik et al., 2024), parthanatos, entotic cell death, neutrophil extracellular trap (NET)otic cell death, lysosome-dependent cell death, autophagy-dependent cell death, immunogenic cell death, cellular senescence, and mitotic catastrophe. However, no comprehensive transcriptomic analysis of these cell death regulators in AD has been conducted (Brokaw et al., 2020; Galluzzi et al., 2018; Goel et al., 2022; Wang et al., 2019). Furthermore, cell death often triggers fibrosis, where fibroblasts replace dead cells and produce extracellular matrix proteins, contributing to fibrous plaque formation in the AD brain (D’Ambrosi and Apolloni, 2020; Yasuma and Gabazza, 2024). Vascular pathology, exacerbated by aging, AD, and vascular dementia, is strongly linked to chronic vascular inflammation and blood vessel dysregulation (Fang et al., 2023), but a comprehensive transcriptomic analysis of fibrosis regulators in AD remains unexplored.

Endoplasmic reticulum (ER) stress, characterized by the accumulation of unfolded or misfolded proteins within the ER, disrupts cellular homeostasis and is implicated in AD progression (Ajoolabady et al., 2022). Our research on ER-related transcriptomic changes demonstrated that the ER acts as an immune organelle (Saaoud et al., 2024), sensing various danger associated molecular patterns (DAMPs) and initiating ER stress that triggers angiotensin II (AngII)-accelerated trained immunity and differential susceptibilities of thoracic and abdominal aortas to aortic aneurysms (Lu et al., 2023). Our study also indicated that the gut microbiota-generated uremic toxin (Sun et al., 2018; Sun et al., 2024) trimethylamine-N-oxide (TMAO) (Chan et al., 2019), induces innate immune memory (also termed trained immunity)(Drummer et al., 2021a; Zhong et al., 2020)) via triggering ER stress, mitochondrial stress, and metabolic reprogramming (Lu et al., 2019; Saaoud et al., 2023). Our recent study proposed that mitochondria act as central immune organelles (Xu et al., 2024), and identified that organelle crosstalk regulators play significant roles in inflammatory diseases and cancer pathogenesis (Liu et al., 2021). We further reported that DNA checkpoint and repair factors act as nuclear sensors for intracellular organelle stresses under both physiological and pathological conditions (Zeng et al., 2018). Moreover, nuclear membrane, nucleolar, and nucleoplasm genes are upregulated in apolipoprotein E deficient (ApoE^–/–^) atherosclerotic aortas and in AngII-induced abdominal aortic aneurysm (AAA) in ApoE^–/–^ mice (Yang et al., 2023). Additionally, procaspase-1 has been observed to translocate to the nucleus in proatherogenic lipid lysophosphatidylcholine (LPC)-activated human aortic endothelial cells (HAECs), where nuclear caspase-1 senses nuclear DAMPs, induces reactive oxygen species (ROS) promoter CYP1B1, and regulates numerous genes involved in HAEC activation and inflammation (Lu et al., 2021). These findings suggest that metabolic reprogramming, which plays crucial role in EndoMT and trained immunity, could be an important mechanism underlying AD pathology (Chen and Holtzman, 2022). Notably, metabolic enhancement through recombinant interferon-γ treatment has been shown to improve glycolytic metabolism and inflammatory responses in microglia, thus mitigating AD pathology in animal models (Baik et al., 2019). Despite these advances, the transcriptomic reprogramming related to organelle stress and bioenergetic metabolism in AD brain remains poorly characterized.

Despite substantial progress, several critical questions remain, highlighting gaps in our knowledge and avenues for further investigation. These questions include whether the transcriptome of the EndoMT pathway is reprogramed in AD, whether the expression of all 14 cell death type regulators remains unchanged in AD, and whether the expression of fibrosis and fibroblast regulators is altered in AD. Using our novel knowledge-based transcriptomic analysis approach, single-cell RNA-seq data from the Single Cell Portal database, and our unpublished data, we have made significant findings. Our research reveals that the 3xTg-AD induces EndoMT, triggers multiple forms of cell death, and promotes fibrosis and fibroblast proliferation in the brain. We have also identified five potential mechanisms—mitochondrial stress, ER stress, nuclear stress, metabolic reprogramming, and catabolic stress—that contribute to EndoMT, cell death, and fibrosis. These mechanistic insights provide new understanding of how AD-related processes are driven by organelle stress and bioenergetic alterations.

## 2 Materials and methods

### 2.1 Animals

Female 3xTg-AD mice (stock no. 34830-JAX) and age-matched C57BL/6J (wild-type, WT) controls were obtained from the Jackson Laboratory (Bar Harbor, ME). To control for variability due to sex and age, only female mice of the same age were used across both genotypes. Each experimental group consisted of three biological replicates (*n* = 3 per group). Mice were housed in a temperature-controlled environment under standard laboratory conditions and maintained on a standard chow diet with ad libitum access to food and water. At 11 months of age, mice were euthanized, and brain tissue samples—including the cortex plus hippocampus— were harvested and immediately processed for RNA-sequencing (RNA-seq) analysis.

### 2.2 RNA-seq and statistical analysis of RNA-seq data

Brain cortex and hippocampus tissues were pooled, homogenized, and lysed in TRIzol reagent (ThermoFisher, GE17-0891-01). RNAs were extracted according to the manufacturer’s protocol then quantified using a Nanodrop (ThermoFisher). The isolated RNA samples were then sent to Genewiz (South Plainfield, NJ) for RNA-Seq analysis. Total RNA libraries were prepared using the Pico Input SMARTer Stranded Total RNA-Seq Kit (Takara). Briefly, 10 ng of total RNA from each sample was reversely transcribed using random priming and reverse transcriptase. Full-length cDNA was synthesized using SMART (Switching Mechanism At the 5’end of RNA Template) technology, which preserves the strand orientation of the RNA. Ribosomal cDNA was hybridized to mammalian-specific R-Probes and cleaved by ZapR. Libraries containing Illumina adapters with TruSeq HT indexes were pooled and loaded onto the Hiseq 2500 platform. Single-end reads of 75 base-pair (bp), with 30 million reads per sample, were generated for subsequent bioinformatic analysis. FASTQ files were mapped to the mouse mm10 genome using the STAR Aligner. Data analysis was performed using the statistical computing environment R, along with the Bioconductor suite of packages and RStudio. Raw data underwent background subtraction, variance stabilization, and normalization via robust spline normalization. Differentially expressed genes were identified using linear modeling and Bayesian statistics with the Limma package. For comparisons between two groups, a two-tailed Student’s *t*-test was used to assess statistical significance.

### 2.3 Transcriptomic data collection

Transcriptomic datasets were obtained from the publicly accessible NIH-NCBI Gene Expression Omnibus (GEO) database.^2^ Relevant datasets were systematically curated and organized for further analysis. Differential gene expression analysis was performed using the GEO2R tool, an interactive web application within the GEO database, which allow comparison between experimental groups and controls using pre-processed data.

### 2.4 Metascape pathway analysis

Pathway enrichment analysis was performed using Metascape^3^, an integrated online tool designed for the functional annotation and interpretation of large-scale omics datasets. Differentially expressed genes (DEGs) identified from RNA-seq data were compiled and uploaded to the Metascape platform to investigate their associated biological pathways, molecular functions, cellular processes, and potential clinical relevance. The analysis incorporated gene ontology (GO) terms, KEGG pathways, Reactome pathways, and other functional categories to provide comprehensive insights into the biological significance of the DEGs.

## 3 Results

### 3.1 The 3xTg-AD model modulates the expression of brain EC vasculature genes, resulting in the upregulation of four specific genes

**FIGURE 1:**
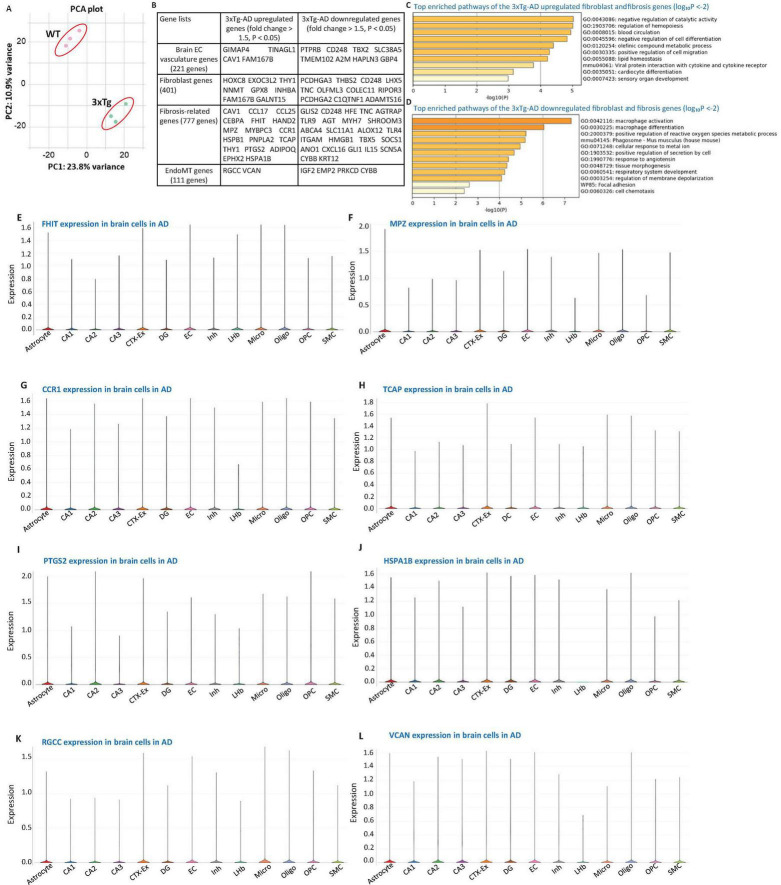
3xTg-AD modulates the expression of brain vasculature genes, fibroblast genes, fibrosis-related genes, and endothelial-to-mesenchymal transition (EndoMT) genes. **(A)** Principal component analysis (PCA) of RNA-seq data demonstrating sample clustering based on genotype. The PCA plot shows clear separation between control (wild-type, WT; pink dots, *n* = 3) and 3xTg-AD (green dots, *n* = 3) samples. Principal component (PC1) and 2 (PC2) explain 23.8% and 10.9% of the variance, respectively. **(B)** Differential gene expression analysis (fold change > 1.5, *P* < 0.05) in 3xTg-AD brains reveals modulation of gene sets associated with brain vasculature (12 genes; four upregulated, eight downregulated), fibroblast (19 genes; eight upregulated, 11 downregulated), fibrosis (41 genes; 17 upregulated, 24 downregulated), and EndoMT (six genes; two upregulated—RGCC and VCAN—and 4 downregulated). **(C,D)** Metascape pathway enrichment analysis of the fibroblast and fibrosis-related gene sets altered in 3xTg-AD highlights key biological pathways implicated in disease pathology (log_10_P < –2). **(E–L)** Single-cell RNA sequencing data depicting expression levels of selected genes in brain cells from AD models: **(E)** FHIT, **(F)** MPZ, **(G)** CCR1, **(H)** TCAP, **(I)** PTGS2, **(J)** HSPA1B, **(K)** RGCC (Regulator of Cell Cycle Protein), and **(L)** VCAN (Versican). Data were obtained from the Single Cell Portal of the Broad Institute (https://singlecell.broadinstitute.org/single_cell/study/SCP1375/integrative-in~situ-mapping-of-single-cell-transcriptional-states-and-tissue-histopathology-in-an-alzheimer-disease-model#study-visualize). Gene sets comprising 221 brain vasculature genes and 401 fibroblast genes were collected from the Human Protein Atlas (HPA) database. The fibrosis-related gene set (777 genes) were compiled from a previous study (PMID: 33519923). EndoMT genes (111 genes) were collected from the HPA database and literature sources (PMIDs: 30864875, 29039786, 30654892).

To investigate whether the 3xTg-AD model induces transcriptomic alteration in brain EC vasculature within the cortex and hippocampus, we analyzed bulk RNA-seq data from brain tissues of 3xTg-AD mice and wild-type (WT) controls, as we recently reported (Saaoud et al., 2025). Differentially expression analysis revealed that 316 genes were significantly upregulated and 412 genes were downregulated in the 3xTg-AD group compared to WT controls, using a cutoff fold change > 1.5 and *p*-value < 0.05, as we previously reported (Saaoud et al., 2025). Principal component analysis (PCA) was conducted to evaluate sample variance and clustering, highlighting distinct transcriptional profiles between the two groups (Figure 1A). A curated list of 221 brain vasculature genes was obtained from the Human Protein Atlas (HPA) database^4^ for targeted analysis. As shown in Figure 1B, 3xTg-AD modulated the expression of brain EC vasculature genes, with four genes significantly upregulated. These include GIMAP4 (GTPase, IMAP family member 4), which promotes immune cell migration (Chen et al., 2022); TINAGL1 (tubulointerstitial nephritis antigen like 1), which promotes EC tube formation (Sato et al., 2022); FAM167B (family with sequence similarity 167 member B), an EC-specific gene (Feng et al., 2019); and CAV1 (caveolin 1), a gene involved in proatherogenic process, leukocyte influx, and CD4^+^ regulatory T cell (Treg) suppression (Engel et al., 2011). Conversely, several genes were significantly downregulated in the 3xTg-AD brain, including PTPRB (which regulates vascular permeability and homeostasis in inflammation) (Vestweber, 2021), CD248 (which induces a maladaptive unfolded protein response in diabetic kidney disease) (Krishnan et al., 2023), TBX2 (which defines a multipotent mesenchymal progenitor pool) (Wojahn et al., 2019), SLC38A5 (which promotes retinal vascular development and pathological neovascularization in a retinopathy model (Wang et al., 2022), TMEM102 [whose overexpression may promote chemoresistance by inhibiting the mitochondria-associated apoptotic pathway (Tai et al., 2022)], Hapln3 (a novel link protein that co-localizes with versican and is upregulated by platelet-derived growth factor in arterial smooth muscle cells) (Ogawa et al., 2004), GBP4 (which serves as a central orchestrator of immune responses to infection, inflammation, and cancer) (Tretina et al., 2019), and A2M (which neutralize cytokines and modifies hemostasis) (Lagrange et al., 2022). These findings suggest that AD brain promotes brain EC activation and inflammatory cell recruitment while inhibiting vascular development and pathological neovascularization.

### 3.2 The 3xTg-AD model modulates the expression of fibroblast- and fibrosis-associated genes, resulting in the upregulation of eight fibroblast-related genes and 17 fibrosis-related genes in brain tissue, as well as six fibrosis-related genes across multiple brain cell types

To determine whether 3xTg-AD induces transcriptomic reprogramming of fibroblasts and fibrosis, we examined the expression changes of 401 fibroblast genes from the HPA database and 777 fibrosis-related genes (Gu et al., 2020) in the 3xTg-AD brain. As shown in Figure 1B, eight out of 401 fibroblast genes and 17 out of 777 fibrosis-related genes were significantly upregulated in the 3xTg-AD brain. Notably, 3xTg-AD also downregulated the expression of eleven fibroblast genes and 24 fibrosis-associated genes. Metascape pathway analysis of the 24 upregulated fibroblast and fibrosis-related genes identified the following key pathways: negative regulation of catalytic activity, regulation of hemopoiesis, blood circulation, negative regulation of cell differentiation, olefinic compound metabolic process, positive regulation of cell migration, lipid homeostasis, viral protein interaction with cytokine and cytokine receptor, and cardiocyte (cardiomyocyte) differentiation. Conversely, the key enriched pathways for the 3xTg-AD downregulated fibroblast and fibrosis-related genes included macrophage activation, macrophage differentiation, positive regulation of ROS metabolic process, phagosome, positive regulation of secretion by cell, response to angiotensin, regulation of membrane depolarization. Among the 17 upregulated fibrosis-related genes, FHIT (fragile histidine triad diadenosine triphosphatase), MPZ (myelin protein zero), CCR1 (C-C motif chemokine receptor 1), TCAP (titin-cap), PTGS2 (prostaglandin-endoperoxide synthase 2), and HSPA1B (heat shock protein family A (Hsp70) member 1B) were found to be expressed in multiple brain cell types, based on data from the Single Cell Portal at the MIT Broad Institute^5^ (Figures 1E–J). Thus, 3xTg-AD model also upregulated 6 fibrosis-related genes in multiple brain cell types in AD.

### 3.3 The 3xTg-AD model modulated the expression of endothelial-to-mesenchymal transition (EndoMT) genes, notably upregulating regulator of cell cycle (RGCC) and versican (VCAN)

To determine whether 3xTg-AD model induces transcriptomic reprogramming in EndoMT, we analyzed the expression of 111 known EndoMT genes (Kovacic et al., 2019; Pardali et al., 2017; Piera-Velazquez and Jimenez, 2019) from the HPA database in our 3xTg-AD RNA-seq dataset. As shown in Figure 1B, 3xTg-AD modulated the expression of six EndoMT genes, with two upregulated genes—RGCC and VCAN—and four downregulated genes. RGCC (response gene to complement 32 protein, RGC-32) is known to regulate cell cycle progression and can be induced by p53 in response to DNA damage or by sublytic levels of complement system proteins, influencing cell cycle activity.^6^ RGCC is also stimulated by growth factors and cytokines, including transforming growth factor β (TGF-β), playing a role in the modulation of processes such as angiogenesis, fibrosis, cell migration, and differentiation (An et al., 2009). Notably, RGCC is essential for promoting the differentiation of T helper 17 (Th17) cells, which are implicated in multiple sclerosis, and in regulating significant transcriptomic changes in astrocytes, favoring the synthesis and secretion of extracellular matrix components, growth factors, axonal growth molecules, and pro-astrogliogenic factors (Tatomir et al., 2022). VCAN (Versican), an extracellular matrix (ECM) proteoglycan, is upregulated alongside other ECM-binding molecules, such as hyaluronan, tumor necrosis factor-α (TNF-α) induced protein 6 (TNFAIP6, TSG-6), and inter-alpha-trypsin inhibitor heavy chain 1 (ITIH1, IαI), during inflammation in various diseases, including cardiovascular, pulmonary, autoimmune diseases, and certain cancers. These interactions form stable scaffolds that act as “landing strips” for inflammatory cells as they migrate from the circulation into tissues (Wight et al., 2020). To validate the roles of RGCC and VCAN in mediating EndoMT in the brain, we examined their expression in multiple brain cell types in AD using single-cell RNA-seq data from the Single Cell Portal database at MIT-Broad Institute^7^, as shown in Figures 1K, L. Our data demonstrated that RGCC is expressed across multiple brain cell types, including ECs and vascular smooth muscle cells (VSMCs). These findings align with recent studies reporting that 14 out of 45 AD risk genes are upregulated within brain vasculature, particularly in brain ECs and VSMCs (Yang et al., 2022). Collectively, our data suggest that 3xTg-AD potentially promotes EndoMT through the upregulation of cytosol/nuclear-localized RGCC and ECM-localized VCAN.

### 3.4 The 3xTg-AD model upregulates several types of cell death—apoptosis, ferroptosis, necroptosis, pyroptosis, programmed necrotic death, and anoikis—in the AD brain

To assess whether the 3xTg-AD model induces transcriptomic reprogramming of cell death pathways, we analyzed the expression changes of 2021 apoptotic cell death genes, based on data from the Mouse Genome Informatics (MGI) database.^8^ As shown in Table 1, the 3xTg-AD model upregulated 43 apoptosis-related genes and downregulated 36. Metascape pathway analysis revealed key pathways associated with the significantly upregulated genes, including the positive regulation of apoptotic process, regulation of apoptotic signaling pathway, regulation of EC proliferation, positive regulation of protein kinase A signaling, regulation of tumor necrosis factor production, regulation of extracellular signal-regulated kinase (ERK)1 and ERK2 cascade, cell activation involved in immune response, regulation of cellular response to stress, regulation of blood vessel EC migration. Conversely, the key pathways associated with downregulated genes included the positive regulation of apoptotic process, glial cell differentiation, positive regulation of cell migration, apoptotic signaling pathway, epithelial cell proliferation, cell chemotaxis, regulation of apoptotic signaling pathway, cellular response to lipid, regulation of cell-cell adhesion, and cellular response to angiotensin.

**TABLE 1:** Differential expression of cell death-related genes in the 3xTg-AD mouse brain.

Gene list/pathways	3xTg-AD upregulated genes (*n* = 316) (fold change > 1.5, *P* < 0.05)	3xTg-AD downregulated genes (*n* = 412) (fold change > 1.5, *P* < 0.05)
Apoptotic cell death genes (2021 genes, Gene ontology)	(43) SDF2L1 CRIP1 DBH DDIT4 EGLN3 FHIT HIF3A PRKD2 SPN TSPO TXNIP APP NET1 GADD45G PLEKHF1 RGCC INHBA LGALS1 ANKRD1 ADIPOQ HOXA5 TCIM VIP EGR3 HSPB1 HSPA5 PAFAH2 RTKN2 HSPB1 PDK4 PLK1 ACAA2 ACTC1 ADA CIDEA DPEP1 ENO1B FFAR4 HAND2 MPZ PTGS2 CAV1 DYNLT1B	(36) PDK1 CD248 TBX2 ACE UACA NTF3 LEF1 PCDHGC4 SOX9 ATP2A3 AGT HMGB2 PRKCD ALOX12 TLR4 ITGAM TMEM102 HMGB1 GNGT1 SOCS1 NGB CCND1 PGLYRP1 COL2A1 TCF7L2 PLA2R1 SFN PCSK9 RPS7 NAIP5 POU4F1 KRT20 CCL21D IRF8 KCNJ11 EMP2
Key enriched pathways log_10_P < −3	•Positive regulation of apoptotic process •Regulation of apoptotic signaling pathway •Regulation of ERK1 and ERK2 cascade •Regulation of endothelial cell proliferation •Positive regulation of protein kinase A signaling •Regulation of small molecule metabolic process •Regulation of tumor necrosis factor production •Negative regulation of catalytic activity •Cell activation involved in immune response •Regulation of cellular response to stress	•Positive regulation of apoptotic process •Glial cell differentiation •Positive regulation of catalytic activity •Positive regulation of cell migration •Epithelial cell proliferation •Apoptotic signaling pathway •Cellular response to lipid •Cell chemotaxis •Positive regulation of epithelial to mesenchymal transition
Ferroptosis genes (564 genes, FerrDB database)	(19) SLC1A5 DPEP1 CDO1 ACSL1 ADIPOQ FABP4 PDK4 PLIN2 HCAR1 HSPB1 CAV1 KIF20A HSPA5 DECR1 PTGS2 PLIN4 NNMT TXNIP DDIT4	(7) ALOX12 TLR4 HMGB1 SOCS1 NGB CYBB AHCY
Key enriched pathways, log_10_P < −3	•PPAR signaling pathway •Regulation of small molecule metabolic process •Negative regulation of catalytic activity •Positive regulation of lipid localization •Response to metal ion •Cell surface receptor protein tyrosine kinase signaling pathway	•Positive regulation of tumor necrosis factor production •positive regulation of apoptotic process
Mitochondrial outer membrane permeabilization involved in programmed cell death genes (36 genes, Gene ontology)	ACAA2	TMEM102
Programmed necrotic cell death genes (25 genes, Gene ontology)	TSPO CAV1	–
MPT-driven necrosis genes (25 genes, PMID: 31355136)	TSPO ATP5G2	HMGB1
NETotic cell death genes (14 genes, PMID: 31355136)	DECR1	–
Mitotic cell death genes (30 genes, PMID: 31355136)	PLK1	–
Anoikis gene (37 genes, PMID: 31355136)	CAV1	–

Transcriptomic analysis of the 3xTg-AD mouse brain revealed significant alterations in the expression of genes involved in multiple regulated cell death pathways. Specifically, 43 apoptosis-related genes, 19 ferroptosis-related genes, one mitochondrial outer membrane permeabilization (MOMP) involved in programmed cell death gene, two programmed necrotic cell death genes, two mitochondrial permeability transition (MPT)-driven necrosis genes, one NETotic cell death gene, one mitotic death gene, and one anoikis gene were upregulated. Concurrently, 36 apoptosis-related genes, seven ferroptosis-related genes, one MOMP-involved in programmed cell death gene, and one MPT-driven necrosis gene were downregulated. Pathway enrichment analysis using Metascape (log_10_P < –3) identified significantly enriched biological pathways associated with these genes. Gene annotations were curated from established resources: apoptosis-, MOMP-, and necrosis-related genes from the Jackson Laboratory Gene Ontology database (http://www.informatics.jax.org/vocab/geneontology/GO:0006915; ferroptosis-related genes from the FerrDB database (http://www.zhounan.org/ferrdb/current/operations/download.html); and MPT-driven necrosis genes, NETotic cell death genes, mitotic cell death genes, and Anoikis genes from PMID: 31355136.

Ferroptosis, a newly identified form of programmed cell death, is triggered by imbalances in iron metabolism, iron-dependent lipid peroxidation, and ROS accumulation (Zhao et al., 2023). Glutathione (GSH) depletion and Glutathione peroxidase 4 (GPX4) inactivation are known to induce ferroptosis (Jiang et al., 2021). To determine whether the 3xTg-AD model affects ferroptosis, we analyzed the expression changes of 264 ferroptosis-related genes, collected from the FerrDB database^9^, among the 3xTg-AD modulated genes. As shown in Table 1, 19 ferroptosis-related genes were upregulated, including solute carrier family 1 member 5 (*SLC1A5)* (Su et al., 2023), *DPEP1*, a dexamethasone-sensitized ferroptosis gene (von Mässenhausen et al., 2022), cysteine dioxygenase type 1 (*CDO1)*, which absorb cysteine competitively, thus restricting the process of GSH synthesis and promoting ferroptosis (Li et al., 2020), and acyl-CoA synthetase long chain family member 1 *(ACSL1)*, which mediate ferroptosis triggered by conjugated linolenic acids (Beatty et al., 2021), and other 15 ferroptosis genes, while downregulated 7 ferroptosis-related genes, including *ALOX12* [the inactivation of arachidonate 12-lipoxygenase, 12S type (*ALOX12*) reduces p53-driven ferroptosis triggered by ROS stress] (Chu et al., 2019), Toll-like receptor 4 (TLR4), high mobility group box 1 (*HMGB1*) [released by anti-inflammatory M2 macrophages, binds to TLR4 on M1 macrophages, leading to the activation of signal transducer and activator of transcription 3 (*STAT3*) signaling in proinflammatory M1 macrophages and promoting the inflammatory response] (Feng et al., 2024), suppressor of cytokine signaling 1 (SOCS1), neuroglobin (NGB), cytochrome B-245 beta chain (*CYBB*), and adenosylhomocysteinase (AHCY). The key pathways of upregulated ferroptosis genes included peroxisome proliferator activated receptor (PPAR) signaling pathway, regulation of small molecule metabolic process, positive regulation of lipid localization, response to metal ion, organic acid catabolic process, cell surface receptor protein tyrosine kinase signaling pathway, however, the downregulated pathways included positive regulation of tumor necrosis factor production and positive regulation of apoptotic process.

Moreover, we identified 299 cell death regulators across 13 different types of cell death pathways, collectively termed the cell death regulatome (Galluzzi et al., 2018; Wang et al., 2019). As shown in Table 1, eight genes were upregulated in six other cell death pathways, including acetyl-CoA acyltransferase 2 *(ACAA2)* in the mitochondrial outer membrane permeability programmed cell death group, translocator protein (*TSPO) and* caveolin 1 *(CAV1)* in the programmed necrotic cell death group, *TSPO* and *ATP5G2* in the mitochondrial permeability transition (MPT)-driven necrosis group, 2,4-dienoyl-CoA reductase 1 (*DECR1)* in the neutrophil extracellular traps (NETotic) cell death group, polo like kinase 1 (*PLK1)* in the mitotic cell death group, and *CAV1* in the anoikis group. Notably, CAV1 was associated with three cell death categories (apoptosis, programmed necrotic cell death, and anoikis), while TSPO was linked to two categories (programmed necrotic cell death and MPT-driven necrosis). Additionally, two genes downregulated in two other cell death pathways, including transmembrane protein 102 (*TMEM102)* in the mitochondrial outer membrane permeability programmed cell death group, and *HMGB1* in the mitochondrial permeability transition (MPT)-driven necrosis group.

The 3xTg-AD brain also downregulated *GBP4* (guanylate binding protein 4) (Figure 1B), which has been shown by us and others to regulate cytosolic recruitment and activation of pro-inflammatory caspase-4 (Drummer et al., 2021b; Wandel et al., 2020). Caspase-4 (human)/caspase-11 (mouse) plays a crucial role in the non-canonical inflammasome activation, leading to inflammatory cell death (pyroptosis) as previously reported (Drummer et al., 2023; Hagar and Miao, 2014; Sun et al., 2024). Our findings suggest that caspase-4/caspase-11 activation in brain cells including ECs may be involved in AD, which was further supported by Wyss-Coray’s team in their 2022 Nature publication (Yang et al., 2022), demonstrating the involvement of microglia and brain ECs in this process. These results indicate that the 3xTg-AD model induces caspase-4/11 activation and pyroptosis in both the AD brain and brain ECs.

Taken together, our findings reveal that the 3xTg-AD brain upregulates 43 apoptosis-related genes, 19 ferroptosis-related genes, one mitochondrial outer membrane permeabilization cell death gene, two programmed necrotic cell death genes, two MPT-driven necrosis genes, one NETotic cell death gene, one mitotic cell death gene, and one anoikis gene. This suggest that the 3xTg-AD model promotes apoptosis, ferroptosis, pyroptosis, and six other types of cell death in the AD brain (Galluzzi et al., 2018; Wang et al., 2019). Additionally, genes such as RGCC (an EndoMT and apoptotic gene), cysteine rich protein 1 (CRIP1, an apoptotic gene), dopamine beta-hydroxylase (DBH, an apoptotic gene), DNA damage-inducible transcript 4 (DDIT4, associated with both apoptosis and ferroptosis), prostaglandin-endoperoxide synthase 2 (PTGS2, involved in both apoptosis and ferroptosis), perilipin 4 (PLIN4, a ferroptotic gene), caspase-4/caspase-11 (casp4, a pyroptotic gene) (Sun et al., 2024), interleukin-1β (IL1β pyroptotic cytokine) (Sun et al., 2024), and TNF-α (TNF-α involved in apoptosis, necroptosis, and trained immunity) (Saaoud et al., 2023; Wang et al., 2019) are expressed in multiple brain cells in AD (Figures 1K, I, and Supplementary Figures 1A–G). The 3xTg-AD model thus demonstrates upregulation of several types of cell death in brain cells, including apoptosis, ferroptosis, necroptosis, pyroptosis, programmed necrotic death, and anoikis.

### 3.5 The 3xTg-AD model induces mitochondrial stress, as evidenced by the upregulation of 32 mitochondrial genes, with a specific induction of mitochondrial stress across multiple cell types in AD brain via the upregulation of six mitochondrial genes

**FIGURE 2:**
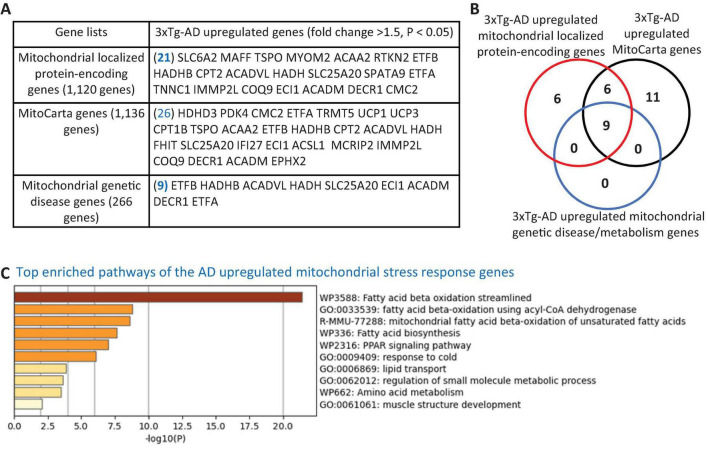
Transcriptional evidence of mitochondrial stress response activation in the 3xTg-AD mouse brain. **(A)** The 3xTg-AD mouse brain exhibit upregulation of 21 mitochondrial-localized protein-encoding genes, 26 nuclear-encoded mitochondrial genes (MitoCarta, Broad Institute), and 9 genes linked to mitochondrial genetic disorders, indicating a potential induction of mitochondrial stress in the AD brain. **(B)** Venn diagram illustrating the overlap among upregulated mitochondrial localized genes, MitoCarta genes, and mitochondrial genetic disease/metabolism genes in the 3xTg-AD brain. **(C)** Metascape pathway analysis reveals significant enrichment of mitochondrial fatty acid beta-oxidation pathways in the 3xTg-AD brain. The 1,120 mitochondrial-localized genes were sourced from the HPA, Mitocarta genes (1,136 nuclear-encoded mitochondrial genes) were retrieved from the Broad Institute (https://www.broadinstitute.org/mitocarta/mitocarta30-inventory-mammalian-mitochondrial-proteins-and-pathways), and a curated list of 266 genes linked to mitochondrial genetic disorder and metabolism were compiled from PMID: 29233888.

To explore the potential mitochondrial mechanisms underlying EndoMT, cell death, and fibrosis in the 3xTg-AD brain, we analyzed transcriptomic reprogramming within the nuclear genome-encoded mitochondrial transcriptome. As shown in Figure 2A, the 3xTg-AD brain upregulated 21 mitochondria-localized protein encoding genes, collected from the HPA database, 26 mitoCarta genes (an inventory of mammalian mitochondrial proteins and pathways, MIT-Broad Institute), and 9 genes associated with mitochondrial genetic diseases and bioenergistic metabolism (Frazier et al., 2019). These upregulated genes overlapped as shown in Figure 2B. Metascape pathway analysis of these upregulated mitochondrial genes identified the top enriched pathways (Figure 2C), including fatty acid (FA) beta oxidation (FAO), FAO using acyl-CoA dehydrogenase, mitochondrial FAO of unsaturated FAs, FA biosynthesis, PPAR signaling pathway, response to cold, lipid transport, regulation of small molecule metabolic process, and muscle structure development. Notably, three mitochondrial localized genes— solute carrier family 6 member 2 (SLC6A2), MAF BZIP transcription factor F (MAFF), troponin C1, slow skeletal and cardiac type (TNNC1)—and three mitoCarta genes— haloacid dehalogenase like hydrolase domain containing 3 (HDHD3), fragile histidine triad diadenosine triphosphatase (FHIT), interferon alpha inducible protein 27 (IFI27)—were upregulated across multiple cell types in AD brain^10^ (Figure 1E and Supplementary Figures 2A–E). In summary, the 3xTg-AD brain induces mitochondrial stress by upregulating 32 mitochondrial genes in brain.

### 3.6 The 3xTg-AD model upregulated EndoMT and cell death pathways in the brain by increasing the expression of nuclear genes, DNA damage response genes, TFs, and differentiation TFs such as FOSB and MEOX1

**FIGURE 3:**
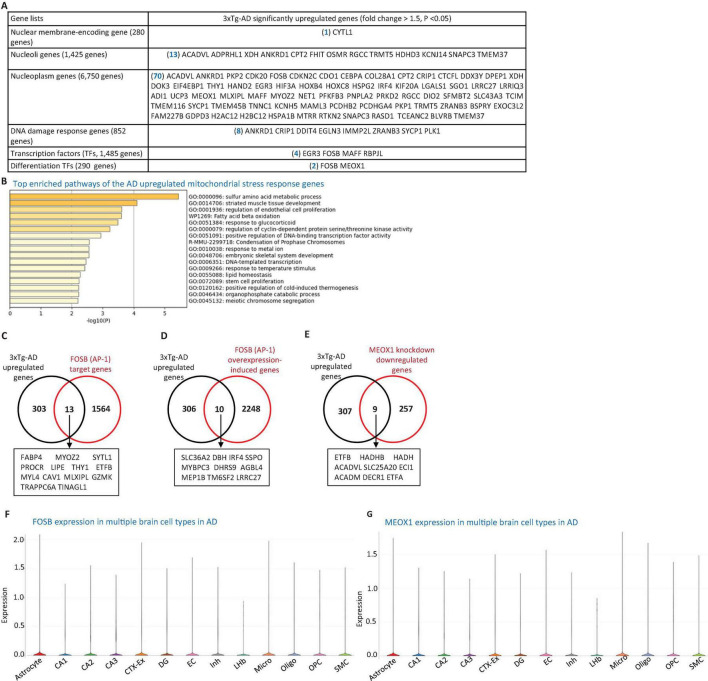
3xTg-AD model induces nuclear stress responses. **(A)** Transcriptomic profiling of the 3xTg-AD brain reveals upregulation of genes associated with nuclear stress, including one nuclear membrane protein gene, 13 nucleolar protein genes, 70 nucleoplasmic protein genes, eight DNA damage response genes, four transcription factors (TFs), and 2 differentiation-associated TFs, suggesting activation of nuclear stress pathways and genomic instability. **(B)** Metascape pathway enrichment analysis of these nuclear-associated genes, including regulation of EC proliferation and fatty acid β-oxidation pathways. **(C,D)** 3xTg-AD-upregulated genes overlap with 13 FOSB target genes and 10 FOSB overexpression-induced genes. **(E)** 3xTg-AD-upregulated genes also intersect with nine genes downregulated by MEOX1 knockdown. **(F,G)** Single-cell RNA-seq data show the expression of FOSB and MEOX1 across multiple brain cell types in AD. Data were collected from the single-cell RNA seq deposited in the Single Cell Portal of the Broad Institute (https://singlecell.broadinstitute.org/single_cell/study/SCP1375/integrative-in~situ-mapping-of-single-cell-transcriptional-states-and-tissue-histopathology-in-an-alzheimer-disease-model#study-visualize). The 280 nuclear membrane-encoding genes, 1,425 nucleoli protein-encoding genes, 6,750 nucleoplasm protein-encoding genes, and 1485 TFs were downloaded from the HPA database. The 852 DNA damage response genes were collected from the MGI Gene Ontology database (https://www.informatics.jax.org/vocab/gene_ontology), and the 290 differentiation TFs were compiled from a previous publication (PMID: 33257861). FOSB target genes were obtained from the MotifMap dataset (https://maayanlab.cloud/Harmonizome/gene_set/AP-1/MotifMap+Predicted+Transcription+Factor+Targets), FOSB overexpression-induced genes from the NCBI GEO database (https://www.ncbi.nlm.nih.gov/geo/query/acc.cgi?acc=GSE111827), and MEOX1 knockdown-downregulated genes from the NCBI GEO database (https://www.ncbi.nlm.nih.gov/geo/geo2r/?acc=GSE133927).

To investigate the potential nuclear stress mechanisms underlying EndoMT, cell death, and fibrosis in the 3xTg-AD brain, we analyzed transcriptomic reprogramming in the nuclear gene transcriptome induced by 3xTg-AD. As shown in Figure 3A, the 3xTg-AD brain upregulated one nuclear membrane protein gene, 13 nucleolar genes, and 70 nucleoplasm genes. Additionally, the 3xTg-AD brain upregulated eight DNA damage response genes, four TFs [early growth response 3 (EGR3), FosB proto-oncogene, AP-1 transcription factor subunit (FOSB), MAFF, and recombination signal binding protein for immunoglobulin kappa J region like (RBPJL)], and two differentiation TFs (Ng et al., 2021), FOSB and mesenchyme homeobox 1 (MEOX1). Metascape pathway analysis of these upregulated nuclear genes revealed the key enriched pathways, including sulfur amino acid metabolic process, muscle tissue development, regulation of EC proliferation, fatty acid beta oxidation, response to glucocorticoids, regulation of cyclin-dependent protein serine/threonine kinase activity, positive regulation of DNA-binding TFs, lipid homeostasis, DNA-templated transcription, and others (Figure 3B).

To determine whether the upregulation of FOSB and MEOX1 contributed to the expression of 316 AD-upregulated genes, we collected FOSB target genes from the MotifMap dataset^11^, FOSB overexpression-induced genes (the dataset ID: GSE111827), and MEOX1 knockdown-downregulated genes (GSE133927) from the NIH-NCBI GEO database^2^. As shown in Figures 3C–E, 13 FOSB target genes, 10 FOSB overexpression-induced genes, and nine MEOX1 knockdown-downregulated genes overlapped with the 3xTg-AD upregulated genes in the brain. To explore the role of FOSB and MEOX1 in 3xTg-AD brain cells, we observed that both TFs were upregulated in multiple cell types in AD brain(see text footnote 10) Figures 3F, G. Notably, two additional TFs—EGR3 (also associated with apoptosis) and MAFF (also linked to mitochondrial-localization)—along with four nucleolar genes, including FHIT (also a mitoCarta gene), Oncostatin M Receptor (OSMR) (also a cellular response to stimulus gene), RGCC (an EndoMT and apoptotic gene), and HDHD3 (a mitoCarta gene), as well as three DNA damage response genes, cysteine rich protein 1 (CRIP1), DNA damage inducible transcript 4 (DDIT4), and Egl-9 family hypoxia inducible factor 3 (EGLN3) (all associated with apoptosis), were also upregulated in AD brain cells, as indicated by the same AD single-cell RNA-seq dataset (Figures 1E, K and Supplementary Figures 1A, C, 2B, D, 3A–C). In summary, these findings demonstrate that the 3xTg-AD brain upregulates EndoMT and cell death pathways in brain cells by enhancing the expression of nuclear genes, DNA damage response genes, TFs, and differentiation TFs FOSB and MEOX1.

### 3.7 The 3xTg-AD model upregulated EndoMT, cell death, and fibrosis in brain cells by increasing the expression of genes associated with cellular stress, oxidative stress, and ER stress

To investigate the potential cellular stress mechanisms driving EndoMT, cell death, and fibrosis in the 3xTg-AD brain, we examined the transcriptomic reprogramming induced by 3xTg-AD related to cellular and oxidative stress as well as ER stress. As shown in Table 2A, 3xTg-AD mice displayed upregulation of 95 genes related to cellular response to stimulus, 32 genes related to cellular response to stress, six genes associated with oxidative stress, and seven ROS regulator genes. Metascape pathway analysis of these upregulated genes (log_10_P < −3) revealed the key enriched pathways, including brown fat cell differentiation, fatty acid metabolic process, PPAR signaling pathway, regulation of small molecule metabolic process, response to hypoxia, regulation of extracellular signal-regulated kinase 1 (ERK1) and ERK2 cascade, positive regulation of lipid localization, lipid homeostasis, cellular response to hormone stimulus, and response to TNF.

**TABLE 2A:** The 3xTg-AD model induces cellular and oxidative stress.

Gene lists	3xTg-AD upregulated genes (fold change > 1.5, *P* < 0.05)	Key enriched pathways (log_10_*P* < −3)
Cellular response to stimulus genes (7,783 genes)	(95) ACAA2 ACSL1 ADA ADIPOQ ALDH1A7 ANKRD1 CAV1 CCL17 CCL25 CDK20 CEBPA CES1D CES1F CIDEA CRIP1 CYP2B9 DBH DDIT4 DPEP1 DYNLT1B EGLN3 EIF4EBP1 ENO1B FABP4 FAM83D FFAR4 FHIT GADD45G GLIPR2 GPD1 HADHB HAND2 HP HSPG2 IMMP2L IRF4 KCNK3 LAT2 LIPE LPL MB MLXIPL MRAP MYOZ2 NET1 OSMR PCK1 PLEKHF1 PLIN2 PLIN5 PRKD2 RGCC SLC2A4 SLC15A2 SPN TCIM TRARG1 TSPAN18 TXNIP UCP1 VIP VMN1R50 XDH APP GPR137B GRPR HCRTR2 HOMER1 MAML3 ZRANB3 BSPRY CCR1 CYP2B19 EGR3 FOSB HSPA5 INHBA MKKS PTGS2 RTKN2 SDF2L1 SYCP1 THY1 ALAD BGLAP3 BLVRB CD14 DOK3 HCAR1 HSPB1 MYH7B PDK4 PLK1 RASD1 SULT1A1	•Brown fat cell differentiation •Fatty acid metabolic process •PPAR signaling pathway •Regulation of small molecule metabolic process •Response to hypoxia •Regulation of ERK1 and ERK2 cascade •Positive regulation of lipid localization •Lipid homeostasis •Cellular response to hormone stimulus •Response to TNF
Cellular response to stress genes (1,728 genes)	(32) ACAA2 ANKRD1 CAV1 CES1D CIDEA CRIP1 DDIT4 EGLN3 EIF4EBP1 ENO1B IMMP2L KCNK3 LIPE MB NET1 PCK1 PLIN2 RGCC SLC2A4 TCIM UCP1 APP ZRANB3 HSPA5 PTGS2 SDF2L1 SYCP1 THY1	
Oxidative stress genes (84 genes)	(6) EHD2 SCD1 TXNIP UCP3 MB PTGS2	
ROS regulator genes (165 genes)	(7) PDK4 PLIN5 TSPO XDH IMMP2L EPHX2 DDIT4	

The 3xTg-AD model upregulates genes associated with cellular stress response, including 95 genes linked to cellular response to stimuli, 32 to cellular response to stress, 6 to oxidative stress, and 7 regulating reactive oxygen species (ROS). Metascape pathway analysis highlights the top functional pathways enriched in these gene sets. Gene lists were retrieved from the Mouse Genome Informatics (MGI) Gene Ontology (GO) database (https://www.informatics.jax.org/vocab/gene_ontology). The oxidative stress genes were compiled from a previous publication (PMID: 21194384), and the ROS regulator genes were obtained from the Gene Set Enrichment Analysis (GSEA) database (https://www.gsea-msigdb.org/gsea/msigdb/cards/HALLMARK_REACTIVE_OXYGEN_SPECIES_PATHWAY).

To explore potential mechanisms by which ER stress contributes to EndoMT, cell death, and fibrosis in the 3xTg-AD brain, we further analyzed transcriptomic reprogramming induced by 3xTg-AD across nine categories of ER stress-related genes. As shown in Table 2B, this analysis revealed: (i) upregulation of five ER-localized protein genes, (ii) upregulation of three ER processing protein genes, (iii) upregulation of four genes involved in the response to ER stress, (iv) upregulation of two AD-promoting genes (Bell et al., 2016) associated with protein kinase R-like ER kinase (EIF2AK3, also known as PERK, gut microbiota generated uremic toxin trimethylamine N-oxide, TMAO (Chan et al., 2019; Saaoud et al., 2023; Sun et al., 2018) receptor (Chen et al., 2019) interaction proteins, (v) upregulation of five genes associated with AD-suppressing (Zhang et al., 2022) activating transcription factor 6 (ATF6) interaction proteins, (vi) upregulation of two genes associated with AD- exacerbating (Duran-Aniotz et al., 2017) endoplasmic reticulum to nucleus signaling 1 (IRE1) interaction proteins, (vii) upregulation of four genes in the ER associated protein degradation (ERAD) pathway (Krshnan et al., 2022), (viii) upregulation of 22 genes induced by the ER stressor thapsigargin (a plant-derived inhibitor of calcium pumps), and (ix) upregulation of 14 genes induced by the ER stressor tunicamycin (an antibiotic produced by the bacterium streptomyces lysosuperificus). Metascape pathway analysis of these upregulated ER stress genes identified the key enriched pathways (log_10_P < −3), including positive regulation of cold-induced thermogenesis, regulation of small molecule metabolic process, PPAR signaling pathway, brown fat cell differentiation, cellular response to hormone stimulus, chaperone cofactor-dependent protein refolding, regulation of carbohydrate metabolic process, metabolism of vitamins and cofactors, triglyceride metabolic process, and regulation of cysteine-type endopeptidase activity involved in apoptotic process.

**TABLE 2B:** The 3xTg-AD model induces ER stress.

Gene lists	3xTg-AD upregulated genes (fold change > 1.5, *P* < 0.05)	Key enriched pathways (log_10_*P* < −3)
ER-localized protein-encoding genes (539 genes)	(5) CYTL1 DBH MRAP VIP STBD1	•Positive regulation of cold-induced thermogenesis •Regulation of small molecule metabolic process •PPAR signaling pathway •Brown fat cell differentiation •Cellular response to hormone stimulus •Chaperone cofactor-dependent protein refolding •Regulation of carbohydrate metabolic process •Metabolism of vitamins and cofactors •Triglyceride metabolic process •Regulation of cysteine-type endopeptidase activity involved in apoptotic process
ER processing protein-encoding genes (170 genes)	(3) HSPA1B HSPA5 UGGT2	
Response to ER stress genes (222 genes)	(4) CAV1 APP HSPA5 SDF2L1	
PERK interaction protein-encoding genes (144 genes)	(2) PCDHGA4 HSPA5	
ATF6 interaction protein-encoding genes (114 genes)	(5) ACAA2 EPHX2 GPIHBP1 NNMT APP	
IRE1 interaction protein-encoding genes (56 genes)	(2) ADIPOQ HSPA5	
ERAD pathway genes (110 genes)	(4) CAV1 UGGT2 HSPA5 SDF2L1	
Thapsigargin-upregulated ER stress genes (1,039 genes) log2FC > 1, *P* < 0.05 (GSE200626)	(22) DDIT4 NNMT SLC6A2 GADD45G GMPPB PRPH XDH ACSL1 CRELD2 GPX8 ANKRD1 EIF4EBP1 UCP1 LAT2 EGR3 PTGS2 FOSB SDF2L1 HSPA5 SYTL1 MAFF DOK3	
Tunicamycin-upregulated ER stress genes (1,817 genes), log2FC > 1, *P* < 0.05 (GSE200626)	(14) CRELD2 GMPPB CPT1B DDIT4 PLIN4 GPR137B APP HSPA5 SDF2L1 MTRR TCEANC2 STBD1 TMEM181C-PS PDK4	

Genes associated with ER stress are upregulated in the 3xTg-AD brain, including five ER-localized protein-encoding genes, three ER processing protein-encoding genes, four response-to-ER stress genes, multiple genes interacting with the three arms of the unfolded protein response (UPR), including two PERK interaction protein-encoding genes, five ATF6 interaction protein-encoding genes, two IRE1 interaction protein-encoding genes. Additional upregulated genes include four ER-associated degradation (ERAD) pathway genes, and genes responsive to the ER stress inducer thapsigargin (22 genes) and tunicamycin (14 genes). Metascape pathway analysis shows the top functional pathways of these upregulated ER stress genes. The ER-localized protein-encoding genes were collected from the HPA database (https://www.proteinatlas.org/search/subcell_location:Endoplasmic+reticulum, https://www.proteinatlas.org/search/subcell_location%3AEndoplasmic+reticulum), ER processing protein-encoding genes from the KEGG pathway database (https://www.genome.jp/dbget-bin/www_bget?pathway:hsa04141), the response-to-ER stress genes from the MGI database (https://www.informatics.jax.org/vocab/gene_ontology/GO:0033554), PERK-interacting protein-encoding genes, ATF6-interacting protein-encoding genes, and IRE1-interacting protein-encoding genes from the NIH database, and ERAD pathway genes from the MGI database (https://www.informatics.jax.org/vocab/gene_ontology/GO:0036503). Additionally, Thapsigargin and tunicamycin-upregulated ER stress genes were retrieved from the NCBI GEO database (GSE200626).

To further elucidate the role of PERK—one of the three key ER stress sensors—in the pathophysiology of AD in the brain of 3xTg mice, we analyzed two transcriptomic datasets from PERK knockout (KO) models, collected from the NCBI GEO database. As shown in Table 2C, the AGBL4 gene was significantly downregulated in the PERK KO group compared to the wild-type (WT) controls. Additionally, 22 genes were downregulated in the PERK KO group following tunicamycin treatment, relative to tunicamycin-treated WT samples. Notably, oxidative stress gene PTGS2 (prostaglandin-endoperoxide synthase 2), DDIT4 (ROS regulator, also upregulated by thapsigargin, tunicamycin, and PERK KO with tunicamycin treatment), and several other ER stress genes upregulated by PERK KO and tunicamycin treatment—such as VCAN (EndoMT gene), eukaryotic translation initiation factor 4E binding protein 1 (EIF4EBP1, cellular stress gene), perilipin 4 (PLIN4), growth arrest and DNA damage inducible gamma (GADD45G), neuronal pentraxin 2 (NPTX2), EGR3—were also upregulated across multiple brain cell types in AD (Figures 1I, L and Supplementary Figures 1C, D, 3A, 4A–C).^10^ Taken together, our findings demonstrate that the 3xTg-AD brain promotes EndoMT, cell death, and fibrosis in the brain and brain cells by upregulating genes related to cellular stress, oxidative stress, and ER stress.

**TABLE 2C:** Transcriptomic evidence for ER stress activation in the 3xTg-AD mouse brain mediated by PERK signaling.

Dataset comparison (GSE29929), log2FC > 1, *P* < 0.05	The number of 3xTg-AD upregulated genes that were downregulated by PERK-KO
PERK-KO (DMSO) compared to WT (DMSO) (393 downregulated genes)	(1) AGBL4
Tunicamycin treated PERK-KO compared to tunicamycin treated WT controls (2010 downregulated genes)	(22) PLIN5 UCP1 VCAN EIF4EBP1 PRKD2 PLIN4 LRRC27 MEM116 HCAR1 GADD45G DDIT4 TRMT5 NPTX2 NAT8F5 HSPA5 MKKS SYTL1 ADI1 SDF2L1 EGR3 STBD1 MTRR

Comparative analysis with liver-specific PERK knockout (KO) models revealed that one 3xTg-AD-upregulated gene was downregulated by PERK deletion in the absence of ER stress, and 22 genes were downregulated following PERK KO under tunicamycin-induced ER stress, suggesting these genes are potentially upregulated via PERK-mediated pathways. PERK KO datasets were collected from the NCBI GEO database (GSE29929).

### 3.8 The 3xTg-AD model drives EndoMT, cell death, and fibrosis in both brain and brain cells through the upregulation of genes involved in lipid metabolism, glucose metabolism, and oxidative phosphorylation (OXPHOS) genes

Mitochondrial abnormalities have been reported in patients with AD, and the roles of full-length APP, and the roles of soluble APPa on mitochondrial bioenergetic metabolism have also been reported (Lopez Sanchez et al., 2019). To investigate the potential metabolic reprogramming mechanisms (via metabolites-induced post-translational modifications of histones) as we reported (Drummer et al., 2021a; Saaoud et al., 2023; Saaoud et al., 2024; Xu et al., 2024) underlying EndoMT, cell death, and fibrosis in the 3xTg-AD brain and brain cells, we examined the transcriptomic changes in metabolic pathways induced by the 3xTg-AD model. As shown in Table 3, we identified the upregulation of 25 lipid and lipoprotein metabolism genes, 8 fatty acid oxidation (FAO) genes, 7 glucose metabolism genes, and 8 OXPHOS genes in the 3xTg-AD brain. Additionally, we observed downregulation of eight lipid and lipoprotein metabolism genes, six glucose metabolism genes, and four OXPHOS genes in the 3xTg-AD brain, which aligns with previous reports of age-dependent declines in mitochondrial complex II activity in a familial AD mouse model (Emmerzaal et al., 2018), as well as reduced cytochrome c oxidase activity in AD (Morais et al., 2021). Metascape pathway analysis of AD-upregulated lipid and lipoprotein metabolism genes identified the key enriched pathways (log_10_P < −3), including fatty acid beta oxidation, metabolism of lipids, PPAR signaling pathway, triacylglyceride synthesis, and lipid localization. The key enriched pathways associated with AD-upregulated FAO genes included metabolism of lipids, negative regulation of catalytic activity, and membrane organization. For AD-upregulated glucose metabolism genes, the top enriched pathways included glucose metabolic process, regulation of small molecule metabolic process, small molecule catabolic process, and regulation of purine nucleotide metabolic process. Notably, several genes—including LPL (lipoprotein lipase, a lipid and lipoprotein metabolism gene), PTGS2 (lipid and lipoprotein metabolism and OXPHOS gene), and APP (amyloid beta precursor protein, OXPHOS gene)—were also upregulated among multiple cell types in AD brain (Figure 1I and Supplementary Figures 5A, B).^10^ Taken together, our findings demonstrate that the 3xTg-AD model promotes EndoMT, cell death, and fibrosis pathways in the brain and brain cells by upregulating genes involved in lipid and lipoprotein metabolism, glucose metabolism, and OXPHOS.

**TABLE 3:** The 3xTg-AD model induces metabolic reprogramming and stress response.

Gene lists/pathways	3xTg-AD upregulated genes (fold change > 1.5, *P* < 0.05)	3xTg-AD downregulated genes (fold change > 1.5, *P* < 0.05)
Lipid and lipoprotein metabolism genes (568 genes)	(25) FABP4 ANKRD1 AGPAT2 CPT1B DPEP1 LPL PLA2G4C LIPE ECI1 CAV1 PNPLA2 PLIN2 GPD1 CYP4B1 HADHB CPT2 ACADVL HADH SLC25A20 ACADM EPHX2 PTGS2 DECR1 ACSL1 HSPG2	(8) SEC24D ABCA1 AGT ALOX12 LPCAT2 A2M CHD9 CYP27B1
Key enriched pathways, log_10_P < −3	•Fatty acid beta oxidation •Metabolism of lipids •PPAR signaling pathway •Triacylglyceride synthesis •Lipid localization •Glycerophospholipid biosynthesis •Long-chain fatty acid metabolic process •Lipid storage •Regulation of lipolysis in adipocytes •Cellular response to tumor necrosis factor	–
fatty acid β-oxidation genes (37 genes)	(8) ACAA2 HADHB ACADVL HADH ECI1 ACADM DECR1 ACSL1	–
Key enriched pathways, log_10_P < −3	•Metabolism of lipids •Negative regulation of catalytic activity •Membrane organization	–
Glucose metabolism related genes (224 genes)	(7) ADIPOQ ENO1B PCK1 PDK4 NNMT GPD1 ACADM	(6) PDK1 IGF2 KCNJ11 C1QTNF1 HMGB1 TCF7L2
Key enriched pathways, log_10_P < −3	•Glucose metabolism process •Regulation of small molecule metabolic process •Positive regulation of small molecule metabolic process •Small molecule catabolic process •Regulation of purine nucleotide metabolic process	–
Oxidative phosphorylation genes (159 genes)	(8) UCP1 ADIPOQ HSPB1 MLXIPL XDH UCP3 PTGS2 APP	(4) PDK1 VRK2 PRKCD TLR4

The 3xTg-AD model exhibit transcriptomic signatures of metabolic dysregulation, including upregulation of 25 genes involved in lipid and lipoprotein metabolism, eight in fatty acid β-oxidation, nine in glucose metabolism, and eight in oxidative phosphorylation (OXPHOS). Additionally, the model shows downregulation of nine glucose metabolism genes and four OXPHOS genes, indicating a shift in energy metabolism and potential mitochondrial stress. Metascape pathway enrichment analysis identified the top functional pathways associated with the upregulated genes related to lipid and lipoprotein metabolism, fatty acid β-oxidation, and glucose metabolism. Gene sets were sourced from: Lipid and lipoprotein metabolism (*n* = 568) Harmonizome 3.0 (https://maayanlab.cloud/Harmonizome/gene_set/Metabolism+of+lipids+and+lipoproteins/Reactome+Pathways+2014), fatty acid β-oxidation MGI Gene ontology (*n* = 37) (https://www.informatics.jax.org/vocab/gene_ontology/GO:0006635), glucose metabolism (*n* = 224) and OXPHOS genes (*n* = 159) Human Protein Atlas database.

### 3.9 The 3xTg-AD model drives EndoMT, cell death, and fibrosis in brain and brain cells through the upregulation of catabolic pathways

Single-cell RNA seq datasets from ischemic stroke, hemorrhagic stroke, and AD models have been integrated to construct metabolic flux profiles at the single-cell level. These three disorders induce shared metabolic shifts in ECs, with altered metabolic modules primarily enriched in transporter-related pathways. These shifts are predicted to potentially reduce metabolites such as pyruvate and fumarate. Additionally, *Lef1* (lymphoid enhancer binding factor 1, *Elk3* (ETS transcription factor), and *Fosl1* (FOS like 1, AP-1 transcription factor subunit) may function as upstream transcriptional regulators driving these metabolic changes (Guo et al., 2023). To explore the potential catabolic reprogramming mechanisms underlying EndoMT, cell death, and fibrosis in the 3xTg-AD brain and brain cells, we examined transcriptomic reprogramming of catabolic process induced by the 3xTg-AD model. As shown in Table 4, we identified the upregulation of 63 catabolic genes and the downregulation of 27 catabolic genes. Metascape pathway analysis of the upregulated catabolic genes revealed the key enriched pathways, including lipid catabolic process, regulation of small molecule metabolic process, glycerolipid catabolic process, PPAR signaling pathway, regulation of lipid localization, positive regulation of cold-induced thermogenesis, mitochondrial FAO of unsaturated fatty acids (FAs), FAO using acyl-CoA dehydrogenase, regulation of sequestering of triglyceride, and regulation of ATP metabolic process. Metascape pathway analysis of the downregulated catabolic genes identified the top five pathways, including carbohydrate derivative catabolic process, small molecule catabolic process, positive regulation of catabolic process, regulation of type II interferon production, and carboxylic acid transport. Notably, six AD-upregulated catabolic genes, including *DBH* (dopamine beta-hydroxylase), *ADA* (adenosine deaminase), *FHIT* (fragile histidine triad diadenosine triphosphatase), *HSPA1B* (heat shock protein family A (Hsp70) member 1B), and VIP (vasoactive intestinal peptide) were upregulated across multiple cell types in AD brain (Supplementary Figure 6).^10^ In conclusion, our findings demonstrate that the 3xTg-AD brain upregulates EndoMT, cell death, and fibrosis pathways in brain and brain cells by enhancing the expressions of catabolic genes to a greater extent than downregulating them.

**TABLE 4:** Differential regulation of catabolic pathways in the 3xTg-AD mouse brain.

Gene lists/pathways	3xTg-AD upregulated genes (fold change > 1.5, *P* < 0.05)	3xTg-AD downregulated genes (fold change > 1.5, *P* < 0.05)
Catabolic genes (2519)	(63) PKP1 ADIPOQ HBB-BT ENO1B HBA-A1 CIDEA STBD1 DBH PCK1 GDPD3 GPIHBP1 CYP2B19 DPEP1 CES1D PLIN5 LPL PLA2G4C PNPLA2 CES1F LIPE TSPO HCAR1 GPD1 ADA FAM83D ACAA2 DDO DIO2 ETFB ALDH1A7 HADHB CPT2 ACADVL CAV1 CYP2B9 HADH MLXIPL UGGT2 CDO1 ALAD PLK1 XDH PAFAH2 PLEKHF1 BLVRB FHIT ECI1 ACADM EPHX2 HSPA5 SDF2L1 DDIT4 ASPG HSPA1B DECR1 MTRR, GPR137B ACAA1B ETFA AGBL4 VIP APP ACSL1	(27) PIPOX RBM38 IRF8 ACE HFE TLR9 SOX9 SYNPO2 PRKCD ENTPD4B TRIM14 ENTPD4 IDUA SLC11A1 FBXO17 HMGB1 TRIM30D HK3 PGLYRP1 BDH2 CYP27B1 PCSK9 CRABP1 RPS7 NANOS2 AHCY SLC6A3
Key enriched pathways log_10_P < −3	•Lipid catabolic process •Regulation of small molecule metabolic process •PPAR signaling pathway •Glycerolipid catabolic process •Regulation of lipid localization •Mitochondrial fatty acid beta-oxidation of unsaturated fatty acids •Fatty acid beta-oxidation using acyl-CoA dehydrogenase •Regulation of sequestering of triglyceride •Regulation of ATP metabolic process •Positive regulation of catabolic process	•Carbohydrate derivative catabolic process •Small molecule catabolic process •Regulation of type II interferon production •Positive regulation of catabolic process •Inflammatory response •Neutrophil degranulation •Negative regulation of catabolic process •Regulation of epithelial cell differentiation

The 3xTg-AD model shows significant transcriptional remodeling of genes involved in catabolic processes, with 63 catabolic genes upregulated and 27 downregulated. Metascape pathway enrichment analysis reveals distinct biological pathways associated with both upregulated and downregulated catabolic genes, suggesting altered degradation and metabolic turnover in the AD model brain. A total of 2,519 catabolic genes were collected from the Mouse Genome Informatics (MGI) Gene Ontology database (https://www.informatics.jax.org/vocab/gene_ontology/GO:0009056).

## 4 Discussion

Despite significant advancement in AD research, current therapies offer no cure and provide only limited palliative benefits against disease progression. AD is characterized by two hallmark pathologies: extracellular plaques composed primarily of Aβ and intraneuronal neurofibrillary tangles (NFTs) primarily composed of hyperphosphorylated Tau protein (DeTure and Dickson, 2019; Trejo-Lopez et al., 2022). However, several pathogenic processes—such as EC activation for inflammatory cell recruitment and EndoMT, cell death, and fibrosis in AD and brain ECs—remain poorly understood.

Using our novel knowledge-based transcriptomic analysis approach (Xu et al., 2021), single-cell RNA-seq data from the Single Cell Portal database at the MIT-Broad Institute, along with our unpublished data, we found compelling evidences that the 3xTg-AD brain induces EndoMT, activates 14 types of cell death, and promotes fibrosis and fibroblast generation in the brain and brain ECs. In addition, we identified five potential mechanisms underlying EndoMT, cell death, and fibrosis, including mitochondrial stress, ER stress, nuclear stress, metabolic reprogramming, and catabolic stress.

In AD, elevated levels of Aβ and p-tau can induce ROS production, leading to excessive mitochondrial fragmentation, fission (Zhu et al., 2013), and defective mitophagy. Five types of mitophagy—primarily focused on neurons—have been reported: (1) Aβ and p-tau-induced mitophagy, (2) stress-induced mitophagy, (3) receptor-mediated mitophagy, (4) ubiquitin-mediated mitophagy, and (5) basal mitophagy (Pradeepkiran and Reddy, 2020). Additionally, sporadic AD exhibits slower mitochondrial dynamics and accumulation of aged mitochondria (Martín-Maestro et al., 2017). Reducing oxidative stress, restoring mitochondrial energetics, and lifestyle interventions (such as diet and exercise) have been shown to decrease Aβ and ameliorate learning and memory impairments in AD (Johri, 2021). Various mitochondria-targeted antioxidants are currently under development for AD (Johri, 2021).

Endoplasmic reticulum stress also plays a distinct role in AD pathogenesis (Ajoolabady et al., 2022), and misfolded proteins and cellular stressors can cause significant structural and molecular alterations in the nucleus (Iatrou et al., 2021). Metabolic dysregulations in AD (Polis and Samson, 2020), including dysfunctional glucose metabolism (Butterfield and Halliwell, 2019), insulin resistance (Pansuria et al., 2012; Sedzikowska and Szablewski, 2021), lipid metabolism abnormalities (Yin, 2023), energy metabolism issues (Yin et al., 2016), gut microbiota dysbiosis (Liu et al., 2020), and hypometabolism (Correas et al., 2024), further contribute to disease progression (Yan et al., 2020). Amino acid oxidation can temporarily compensate for decreased glucose metabolism via glutaminolysis-fed tricarboxylic acid (TCA) cycle(Xu et al., 2023; Xu et al., 2024), but altered levels of amino acids and their catabolites(Sun et al., 2018) may eventually lead to toxicities that exacerbate AD progression (Griffin and Bradshaw, 2017). Collectively, these findings strongly support our identification of five mechanisms underlying EndoMT, cell death, and fibrosis in the AD brain and brain ECs.

**FIGURE 4:**
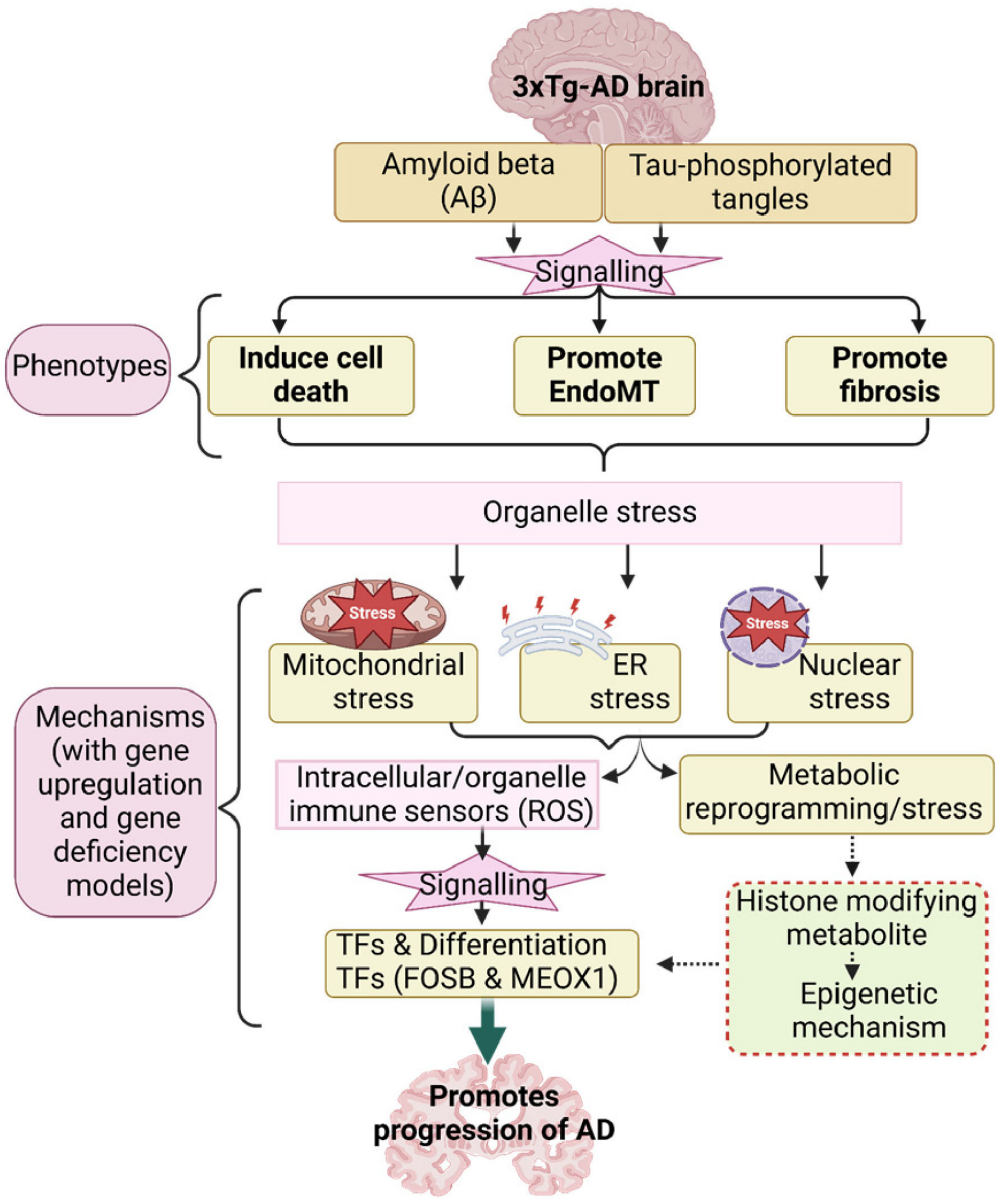
Our working model.

Based on our results, we propose a new working model (Figure 4). *First*, the 3xTg-AD brain induces expression of genes associated with several types of cell death, exceeding those previously reported (Goel et al., 2022). This observation aligns with findings that neurons in AD brains undergo a programmed form of cell death known as necroptosis in response to amyloid plaques and tau tangles, which are misfolded proteins (Goel et al., 2022). *Second*, the 3xTg-AD brain promotes EndoMT in brain ECs and fibroblasts, as well as fibrosis in the AD brain. In line with this, our previous report identified AD as an auto-innate immune/auto-inflammatory and autoimmune disease (Saaoud et al., 2025). Our findings on increased EndoMT in AD brain ECs support current reports suggesting that neuro-inflammatory diseases disrupt the blood-brain/CNS barrier (BBB) through crosstalk between pro-inflammatory and EndoMT signaling pathways (Sun et al., 2022), and that microvascular contributions to AD pathogenesis and endotheliopathy are significant features of the disease (Tarawneh, 2023). *Third*, leveraging our knowledge-based transcriptomic analysis approach (Xu et al., 2021), single-cell RNA-seq data, and our unpublished data, we found robust evidence of five mechanisms underlying EndoMT, cell death, and fibrosis in AD brain and brain ECs. These mechanisms include mitochondrial stress, ER stress, nuclear stress, metabolic reprogramming, and catabolic stress. *Fourth*, mechanistic analysis revealed that genes upregulated by 3xTg-AD were also upregulated by FOSB overexpression while downregulated by deficiencies in MEOX1 and ER stress kinase EIF2AK3 (PERK) knockdown, suggesting that these master genes may drive AD pathology and associated mechanisms. Overall, our panoramic and mechanistic findings provide novel insights into the roles of 3xTg AD-induced endotheliopathy, EndoMT, cell death, and fibrosis in promoting AD cerebrovascular damage and disease progression via organelle stresses and bioenergetic mechanisms.

## Data Availability

Original datasets are available in a publicly accessible repository: GSE305112. The original contributions presented in the study are publicly available. This data can be found here: [https://www.ncbi.nlm.nih.gov/geo/query/acc.cgi?acc=GSE305112].

## Footnotes

1
https://www.alz.org/alzheimer_s_dementia

2
https://www.ncbi.nlm.nih.gov/gds

3
https://metascape.org

4
https://www.proteinatlas.org/

5
https://singlecell.broadinstitute.org/single_cell/study/SCP1375/integrative-in~situ-mapping-of-single-cell-transcriptional-states-and-tissue-histopathology-in-an-alzheimer-disease-model

6
https://www.ncbi.nlm.nih.gov/gene/28984

7
https://singlecell.broadinstitute.org/single_cell

8
https://informatics.jax.org/

9
http://www.zhounan.org/ferrdb/current/

10
https://singlecell.broadinstitute.org/single_cell/study/SCP1375/integrative-in-situ-mapping-of-single-cell-transcriptional-states-and-tissue-histopathology-in-an-alzheimer-disease-model#study-visualize

11
https://maayanlab.cloud/Harmonizome/gene_set/AP-1/MotifMap+Predicted+Transcription+Factor+Targets
