# Supplementary material for: Organelle stresses and energetic metabolisms promote endothelial–to–mesenchymal transition and fibrosis via upregulating FOSB and MEOX1 in Alzheimer’s disease
Source: Front Mol Neurosci. 2025 Aug 22;18:1605012. doi: 10.3389/fnmol.2025.1605012 (PMC12411492; doi:10.3389/fnmol.2025.1605012)
Supplement: Supplementary file 1 [file Presentation_1.pptx]

## Slide 1
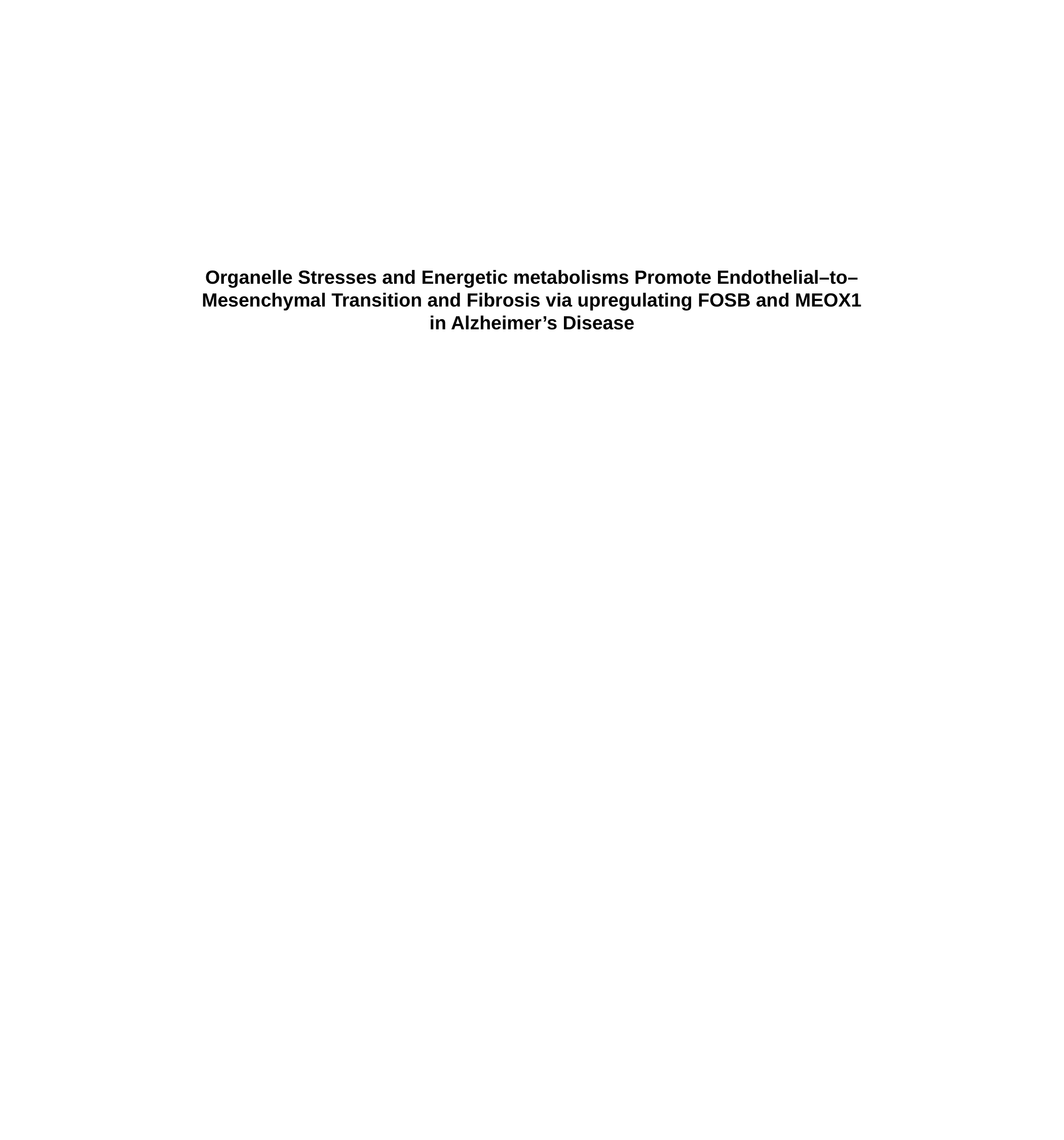

Organelle Stresses and Energetic metabolisms Promote Endothelial–to– Mesenchymal Transition and Fibrosis via upregulating FOSB and MEOX1 in Alzheimer’s Disease

## Slide 2
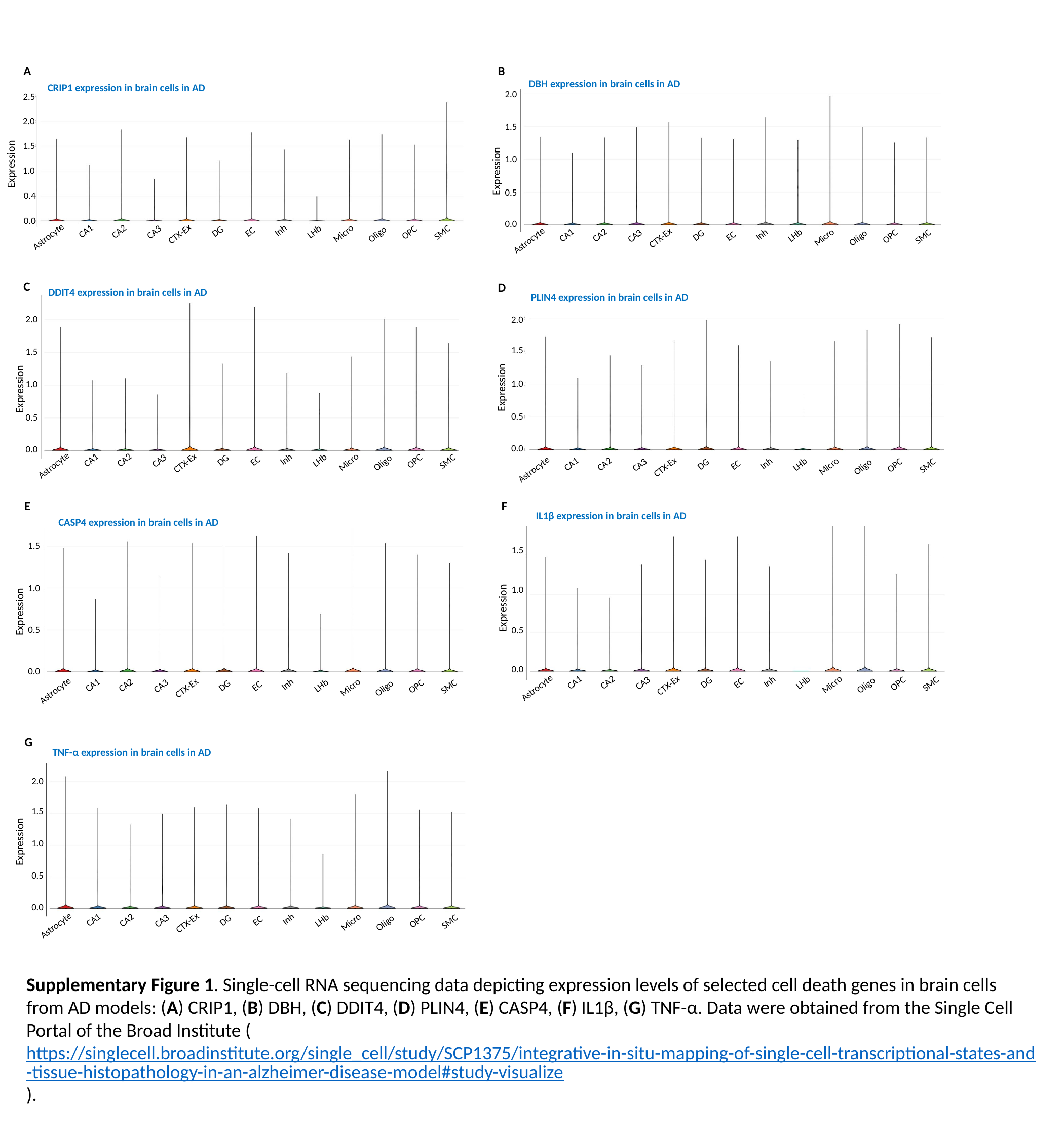

A
B
DBH expression in brain cells in AD
CRIP1 expression in brain cells in AD
2.0
1.5
1.0
0.5
0.0
2.5
2.0
1.5
1.0
0.4
0.0
Expression
Expression
CA2
Inh
OPC
CA1
DG
EC
CA3
LHb
SMC
Micro
Oligo
CTX-Ex
Astrocyte
CA2
Inh
OPC
CA1
DG
EC
CA3
LHb
SMC
Micro
Oligo
CTX-Ex
Astrocyte
C
DDIT4 expression in brain cells in AD
2.0
1.5
1.0
0.5
0.0
Expression
CA2
Inh
OPC
CA1
DG
EC
CA3
LHb
SMC
Micro
Oligo
CTX-Ex
Astrocyte
D
PLIN4 expression in brain cells in AD
2.0
1.5
1.0
0.5
0.0
Expression
CA2
Inh
OPC
CA1
DG
EC
CA3
LHb
SMC
Micro
Oligo
CTX-Ex
Astrocyte
E
CASP4 expression in brain cells in AD
1.5
1.0
0.5
0.0
Expression
CA2
Inh
OPC
CA1
DG
EC
CA3
LHb
SMC
Micro
Oligo
CTX-Ex
Astrocyte
F
IL1β expression in brain cells in AD
1.5
1.0
0.5
0.0
Expression
CA2
Inh
OPC
CA1
DG
EC
CA3
LHb
SMC
Micro
Oligo
CTX-Ex
Astrocyte
G
TNF-α expression in brain cells in AD
2.0
1.5
1.0
0.5
0.0
Expression
CA2
Inh
OPC
CA1
DG
EC
CA3
LHb
SMC
Micro
Oligo
CTX-Ex
Astrocyte
Supplementary Figure 1. Single-cell RNA sequencing data depicting expression levels of selected cell death genes in brain cells from AD models: (A) CRIP1, (B) DBH, (C) DDIT4, (D) PLIN4, (E) CASP4, (F) IL1β, (G) TNF-α. Data were obtained from the Single Cell Portal of the Broad Institute (https://singlecell.broadinstitute.org/single_cell/study/SCP1375/integrative-in-situ-mapping-of-single-cell-transcriptional-states-and-tissue-histopathology-in-an-alzheimer-disease-model#study-visualize).

## Slide 3
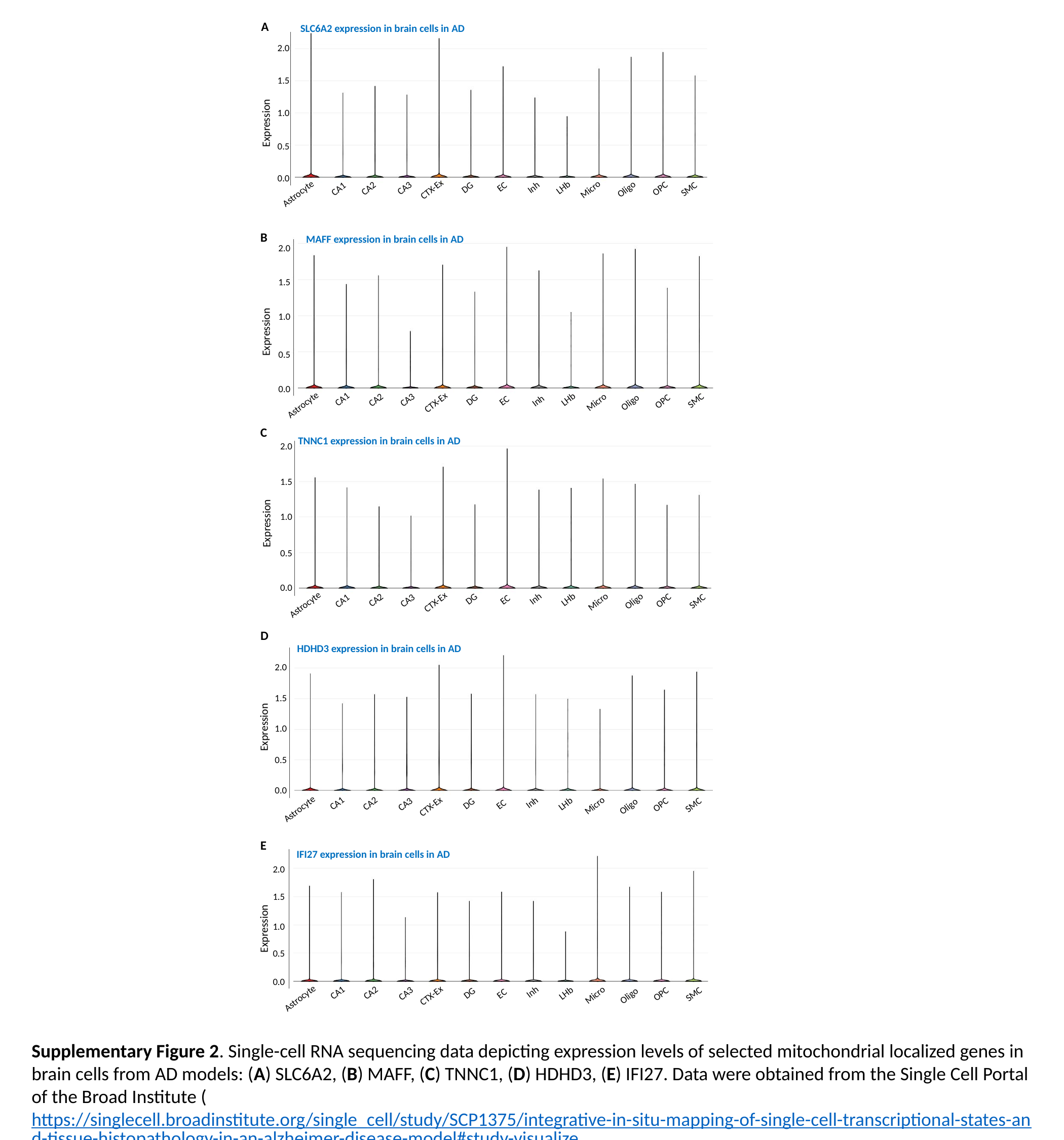

A
SLC6A2 expression in brain cells in AD
2.0
1.5
1.0
0.5
0.0
Expression
EC
OPC
LHb
CA3
CA2
Inh
DG
SMC
CA1
Oligo
Micro
CTX-Ex
Astrocyte
B
MAFF expression in brain cells in AD
2.0
1.5
1.0
0.5
0.0
Expression
CA2
CA3
CA1
SMC
LHb
DG
OPC
EC
Inh
Micro
Oligo
CTX-Ex
Astrocyte
C
TNNC1 expression in brain cells in AD
Expression
2.0
1.5
1.0
0.5
0.0
Inh
EC
DG
OPC
CA2
LHb
CA3
SMC
CA1
Oligo
Micro
CTX-Ex
Astrocyte
D
HDHD3 expression in brain cells in AD
2.0
1.5
1.0
0.5
0.0
Expression
CA2
Inh
OPC
CA1
DG
EC
CA3
LHb
SMC
Micro
Oligo
CTX-Ex
Astrocyte
E
IFI27 expression in brain cells in AD
2.0
1.5
1.0
0.5
0.0
Expression
CA2
Inh
OPC
CA1
DG
EC
CA3
LHb
SMC
Micro
Oligo
CTX-Ex
Astrocyte
Supplementary Figure 2. Single-cell RNA sequencing data depicting expression levels of selected mitochondrial localized genes in brain cells from AD models: (A) SLC6A2, (B) MAFF, (C) TNNC1, (D) HDHD3, (E) IFI27. Data were obtained from the Single Cell Portal of the Broad Institute (https://singlecell.broadinstitute.org/single_cell/study/SCP1375/integrative-in-situ-mapping-of-single-cell-transcriptional-states-and-tissue-histopathology-in-an-alzheimer-disease-model#study-visualize).

## Slide 4
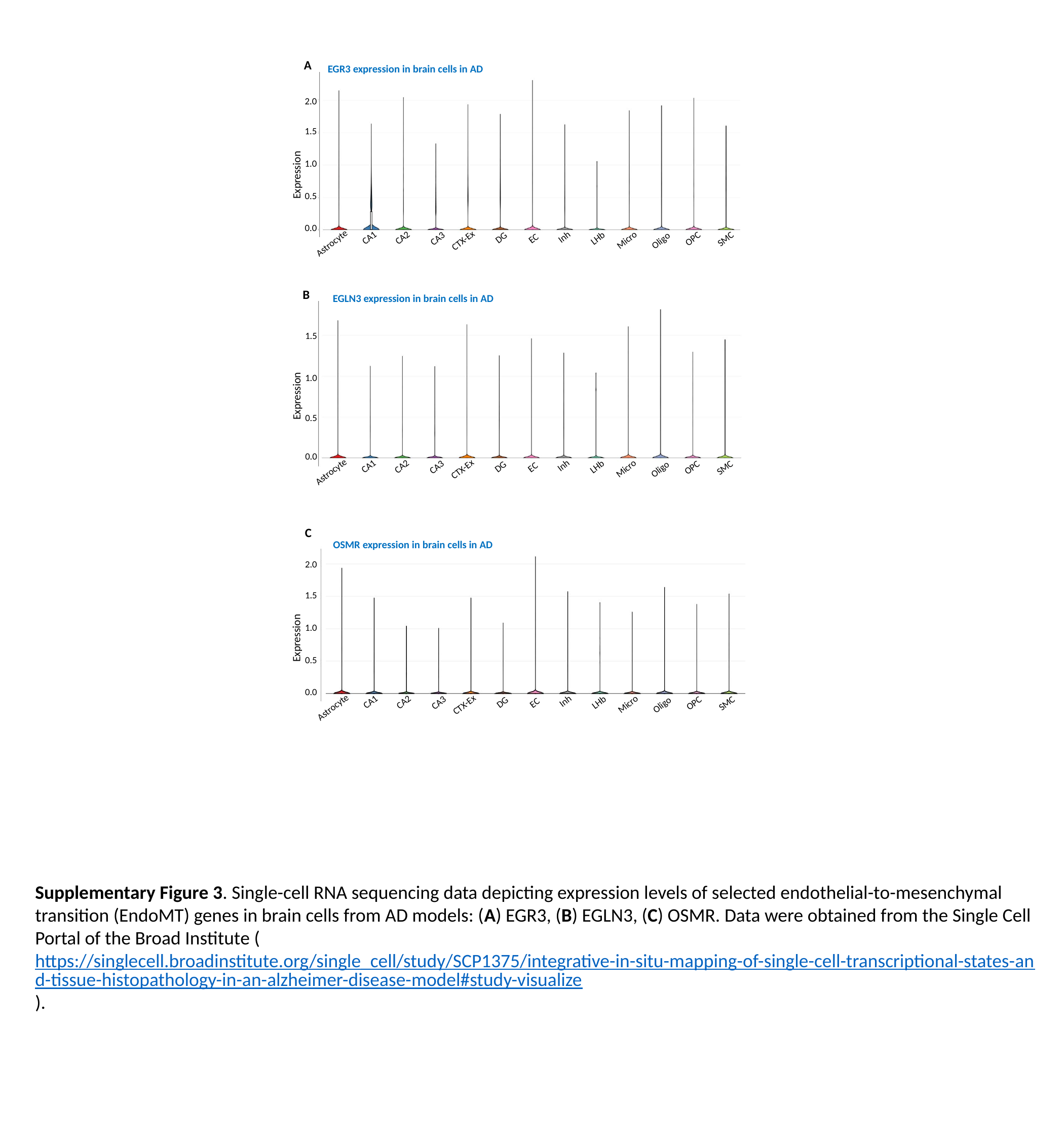

A
EGR3 expression in brain cells in AD
2.0
1.5
1.0
0.5
0.0
Expression
CA2
Inh
OPC
CA1
DG
EC
CA3
LHb
SMC
Micro
Oligo
CTX-Ex
Astrocyte
B
EGLN3 expression in brain cells in AD
1.5
1.0
0.5
0.0
Expression
CA2
Inh
OPC
CA1
DG
EC
CA3
LHb
SMC
Micro
Oligo
CTX-Ex
Astrocyte
C
OSMR expression in brain cells in AD
2.0
1.5
1.0
0.5
0.0
Expression
CA2
Inh
OPC
CA1
DG
EC
CA3
LHb
SMC
Micro
Oligo
CTX-Ex
Astrocyte
Supplementary Figure 3. Single-cell RNA sequencing data depicting expression levels of selected endothelial-to-mesenchymal transition (EndoMT) genes in brain cells from AD models: (A) EGR3, (B) EGLN3, (C) OSMR. Data were obtained from the Single Cell Portal of the Broad Institute (https://singlecell.broadinstitute.org/single_cell/study/SCP1375/integrative-in-situ-mapping-of-single-cell-transcriptional-states-and-tissue-histopathology-in-an-alzheimer-disease-model#study-visualize).

## Slide 5
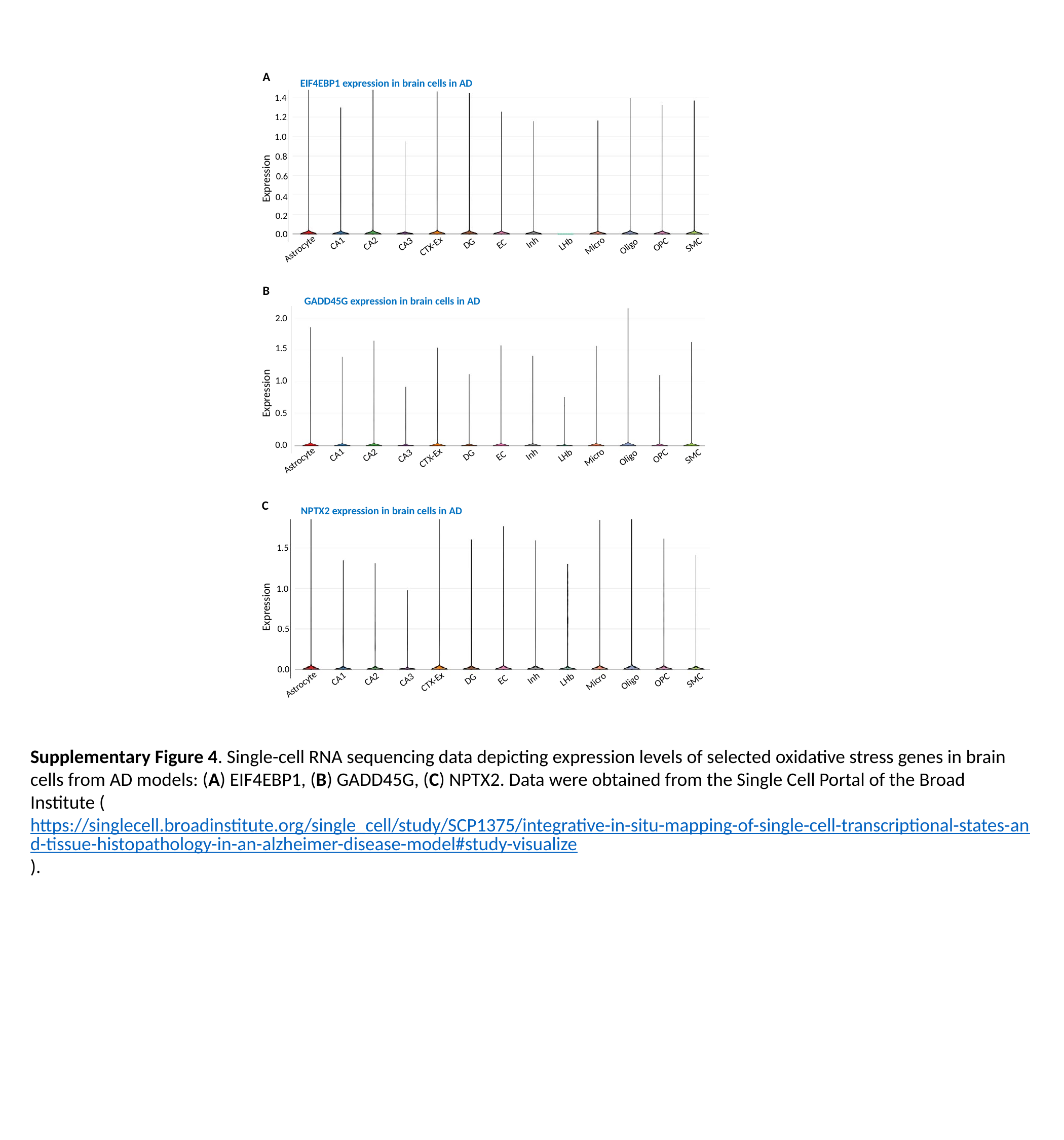

A
EIF4EBP1 expression in brain cells in AD
1.4
1.2
1.0
0.8
0.6
0.4
0.2
0.0
Expression
CA2
Inh
OPC
CA1
DG
EC
CA3
LHb
SMC
Micro
Oligo
CTX-Ex
Astrocyte
B
GADD45G expression in brain cells in AD
2.0
1.5
1.0
0.5
0.0
Expression
CA2
Inh
OPC
CA1
DG
EC
CA3
LHb
SMC
Micro
Oligo
CTX-Ex
Astrocyte
C
NPTX2 expression in brain cells in AD
1.5
1.0
0.5
0.0
Expression
CA2
Inh
OPC
CA1
DG
EC
CA3
LHb
SMC
Micro
Oligo
CTX-Ex
Astrocyte
Supplementary Figure 4. Single-cell RNA sequencing data depicting expression levels of selected oxidative stress genes in brain cells from AD models: (A) EIF4EBP1, (B) GADD45G, (C) NPTX2. Data were obtained from the Single Cell Portal of the Broad Institute (https://singlecell.broadinstitute.org/single_cell/study/SCP1375/integrative-in-situ-mapping-of-single-cell-transcriptional-states-and-tissue-histopathology-in-an-alzheimer-disease-model#study-visualize).

## Slide 6
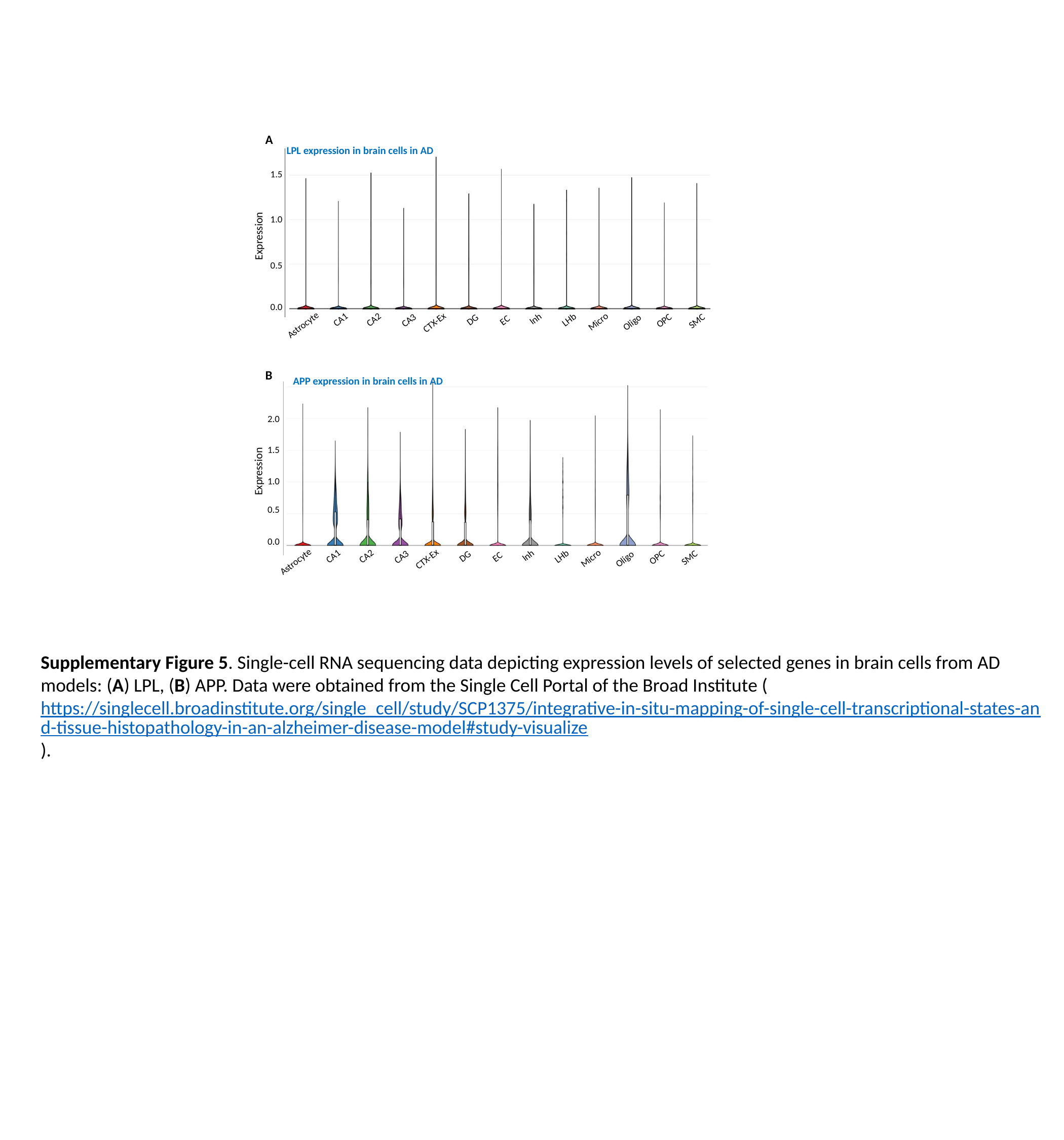

A
LPL expression in brain cells in AD
Expression
1.5
1.0
0.5
0.0
CA2
Inh
OPC
CA1
DG
EC
CA3
LHb
SMC
Micro
Oligo
CTX-Ex
Astrocyte
B
APP expression in brain cells in AD
Expression
2.0
1.5
1.0
0.5
0.0
CA2
Inh
OPC
CA1
DG
EC
CA3
LHb
SMC
Micro
Oligo
CTX-Ex
Astrocyte
Supplementary Figure 5. Single-cell RNA sequencing data depicting expression levels of selected genes in brain cells from AD models: (A) LPL, (B) APP. Data were obtained from the Single Cell Portal of the Broad Institute (https://singlecell.broadinstitute.org/single_cell/study/SCP1375/integrative-in-situ-mapping-of-single-cell-transcriptional-states-and-tissue-histopathology-in-an-alzheimer-disease-model#study-visualize).

## Slide 7
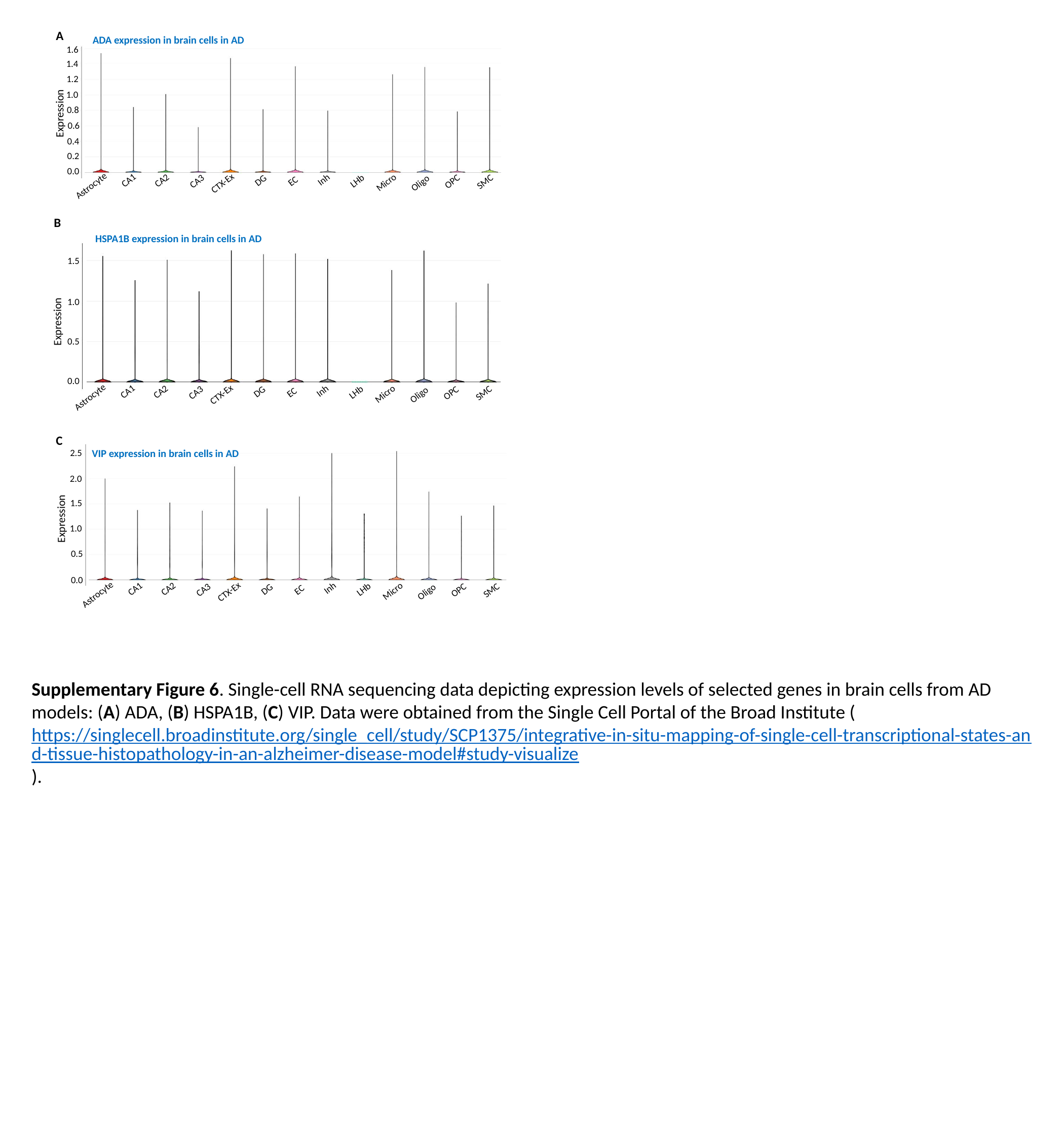

A
ADA expression in brain cells in AD
1.6
1.4
1.2
1.0
0.8
0.6
0.4
0.2
0.0
Expression
CA2
Inh
OPC
CA1
DG
EC
CA3
LHb
SMC
Micro
Oligo
CTX-Ex
Astrocyte
B
HSPA1B expression in brain cells in AD
1.5
1.0
0.5
0.0
Expression
CA2
Inh
OPC
CA1
DG
EC
CA3
LHb
SMC
Micro
Oligo
CTX-Ex
Astrocyte
C
VIP expression in brain cells in AD
Expression
2.5
2.0
1.5
1.0
0.5
0.0
CA2
Inh
OPC
CA1
DG
EC
CA3
LHb
SMC
Micro
Oligo
CTX-Ex
Astrocyte
Supplementary Figure 6. Single-cell RNA sequencing data depicting expression levels of selected genes in brain cells from AD models: (A) ADA, (B) HSPA1B, (C) VIP. Data were obtained from the Single Cell Portal of the Broad Institute (https://singlecell.broadinstitute.org/single_cell/study/SCP1375/integrative-in-situ-mapping-of-single-cell-transcriptional-states-and-tissue-histopathology-in-an-alzheimer-disease-model#study-visualize).
